# A Kinase Interacting Protein 1 (AKIP1) promotes cardiomyocyte elongation and physiological cardiac remodelling

**DOI:** 10.1038/s41598-023-30514-1

**Published:** 2023-03-10

**Authors:** Kirsten T. Nijholt, Pablo I. Sánchez-Aguilera, Harmen G. Booij, Silke U. Oberdorf-Maass, Martin M. Dokter, Anouk H. G. Wolters, Ben N. G. Giepmans, Wiek H. van Gilst, Joan H. Brown, Rudolf A. de Boer, Herman H. W. Silljé, B. Daan Westenbrink

**Affiliations:** 1 Department of Cardiology, University Medical Centre Groningen, University of Groningen, P.O. Box 30.001, Hanzeplein 1, 9713 GZ, 9700 RB Groningen, The Netherlands; 2 Department of Biomedical Sciences of Cells and Systems, University Medical Centre Groningen, University of Groningen, Groningen, The Netherlands; 3 Department of Pharmacology, University of California San Diego, La Jolla, USA

**Keywords:** Cardiology, Molecular medicine

## Abstract

A Kinase Interacting Protein 1 (AKIP1) is a signalling adaptor that promotes physiological hypertrophy in vitro. The purpose of this study is to determine if AKIP1 promotes physiological cardiomyocyte hypertrophy in vivo. Therefore, adult male mice with cardiomyocyte-specific overexpression of AKIP1 (AKIP1-TG) and wild type (WT) littermates were caged individually for four weeks in the presence or absence of a running wheel. Exercise performance, heart weight to tibia length (HW/TL), MRI, histology, and left ventricular (LV) molecular markers were evaluated. While exercise parameters were comparable between genotypes, exercise-induced cardiac hypertrophy was augmented in AKIP1-TG *vs.* WT mice as evidenced by an increase in HW/TL by weighing scale and in LV mass on MRI. AKIP1-induced hypertrophy was predominantly determined by an increase in cardiomyocyte length, which was associated with reductions in p90 ribosomal S6 kinase 3 (RSK3), increments of phosphatase 2A catalytic subunit (PP2Ac) and dephosphorylation of serum response factor (SRF). With electron microscopy, we detected clusters of AKIP1 protein in the cardiomyocyte nucleus, which can potentially influence signalosome formation and predispose a switch in transcription upon exercise. Mechanistically, AKIP1 promoted exercise-induced activation of protein kinase B (Akt), downregulation of CCAAT Enhancer Binding Protein Beta (C/EBPβ) and de-repression of Cbp/p300 interacting transactivator with Glu/Asp rich carboxy-terminal domain 4 (CITED4). Concludingly, we identified AKIP1 as a novel regulator of cardiomyocyte elongation and physiological cardiac remodelling with activation of the RSK3-PP2Ac-SRF and Akt-C/EBPβ-CITED4 pathway. These findings suggest that AKIP1 may serve as a nodal point for physiological reprogramming of cardiac remodelling.

## Introduction

The heart continuously adapts to fluctuations in metabolic demands of peripheral tissues^1,2^. In response to sustained or repetitive increases in workload such as exercise or pressure overload, the heart responds by increasing muscle mass, a process known as cardiac hypertrophy^1^. The increase in muscle mass provides mechanical advantages as it reduces ventricular wall stress and increases contractile performance^1,3^. Although cardiac hypertrophy provides early functional compensation, it can also be characterised by maladaptive changes in the histological, molecular, biochemical, and metabolic composition of the myocardium that can ultimately cause heart failure (HF)^1,3,4^.

There are, however, important distinctions between cardiac hypertrophy that occur in response to physiological stimuli such as exercise and pregnancy and pathological stimuli such as pressure overload^1,2^. Most importantly, physiological and pathological cardiac hypertrophy are governed by distinct signal transduction pathways with distinct histological, biochemical and molecular signatures. For instance, pathological cardiac hypertrophy is accompanied by a predominant increase in cardiomyocyte width, relative reductions in capillary density, re-expression of the foetal cardiac genes, mitochondrial dysfunction, and fibrosis^1,3–5^. In contrast, physiological cardiac hypertrophy induced by endurance exercise is characterized by a preferential increase in cardiomyocyte length (eccentric hypertrophy), expansion of the capillary network and improvements in mitochondrial quantity and quality^1,2,4,6–8^.

While intense efforts have been devoted to the identification of pathways underlying pathological hypertrophy, relatively little is known about physiological hypertrophy. Identifying key factors regulating physiological cardiac hypertrophy should allow the design of therapies to shift pathological hypertrophy towards a more physiological end of the spectrum.

An important distinction between physiological and pathological hypertrophy is that they are governed by distinct growth factors and signal transduction pathways^1^. Our group recently identified the signalling adaptor protein A Kinase Interacting Protein 1 (AKIP1) as a pro-hypertrophic gene^9^, the myocardial expression of which is increased in response to exercise^10^ as well as in a transgenic mouse model of physiological hypertrophy^11^. In cultured cardiomyocytes, overexpression of AKIP1 improved mitochondrial function^12^ and induced a physiological type of hypertrophy through activation of the Akt-mTOR pathway^13^. Accordingly, we tested the hypothesis that cardiomyocyte-specific overexpression of AKIP1 in mice promotes cardiac hypertrophy in response to voluntary exercise in vivo.

## Methods

### Ethical approval

The Animal Ethics Committee from the University of Groningen approved the animal experiments (DEC6237F, 199105-01-005), which were performed following the protocols from Directive 2010/63/EU of the European Parliament. The study was reported in accordance with the recommendations of the ARRIVE guidelines.

### Animal model

A total of 44 adult male mice (8–12 weeks old) were included in the study population. This study population included mice with cardiomyocyte-specific overexpression of AKIP1 (AKIP1-TG) and their wild type (WT) littermates. AKIP1-TG mice were generated as described previously^14^. To validate whether there was AKIP1 overexpression in the heart of AKIP1-TG mice, mRNA and Western blot analysis was performed (Supplementary Fig. S1).

### Experimental model

**Figure 1:**
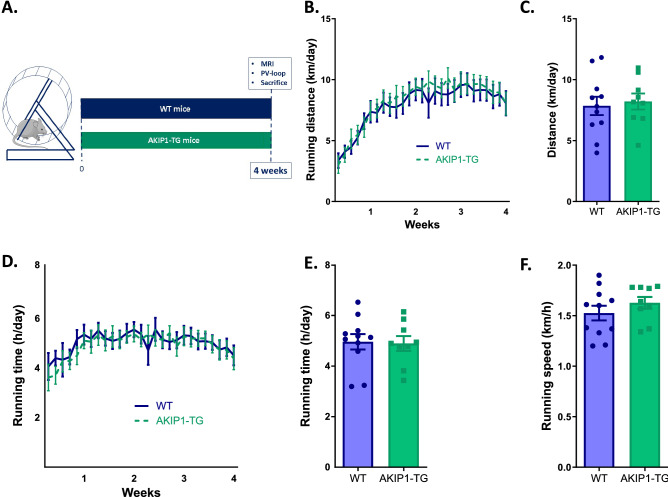
Exercise parameters during four-week period of voluntary wheel running, (N = 9–11/group). Shown are, (**A**) experimental design including voluntary wheel running protocol, (**B**) daily running distance in kilometres per day (km/day), (**C**) average daily running distance in kilometres per day (km/day), (**D**) running time in hours per day (h/day), (**E**) average running time in hours per day (h/day), and (**F**) average speed in kilometres per hour (km/h). Mice included were aged 8–12 weeks old. WT = wild type mice, AKIP1-TG = AKIP1 transgenic mice. Graphs represent mean ± standard error of the mean (SEM). Statistical analysis was performed with Student’s *t-*test, < 0.05 was considered statistically significant. Part of the illustration in panel (**A**) contains images from Servier Medical Art by Servier, licensed under a Creative Commons Attribution 3.0 unported license.

AKIP1-TG mice and their WT littermates were caged individually in the presence or absence of a running wheel for four weeks. This resulted in two running groups (WT Run, AKIP1-TG Run) and two sedentary groups (WT Sed, AKIP1-TG Sed) (Fig. 1A). All mice had ad libitum access to food and water and were housed in 12:12 h dark–light cycles.

### Exercise performance

Exercise performance was recorded using a cyclometer connected to the running wheel^15^. Mice that were housed with a running wheel in their individual cage, had a sensor and cyclometer attached to the running wheel. The sensor on the running wheel automatically detected running activity as soon as mice started performing exercise. Using the sensor and cyclometer, we were able to record exercise parameters including running speed, distance and time. To monitor and determine exercise capacity, the cyclometer was read out on a daily basis, every 24 h.

### Cardiac function parameters and haemodynamics

After completion of the running wheel protocol, cardiac magnetic resonance imaging (MRI) using 9.4 T 400 MR system (Bruker Biospin, Ellingen, Germany) was performed as previously described^14^. Manual analysis was performed using Circle Cardiovascular Imaging (CCI, version 42, Canada) software. Thereafter, aortic and intracardiac pressure parameters were obtained using a pressure volume system with a Millar catheter (Micro-Tip 1.4 French; SPR, -839, Millar Instruments, USA) as performed before^16^. Analysis was performed with Labchart 7 software (ADInstruments, LtD, Dunedin, New Zealand). Cardiac function was determined under anaesthesia of 2% isoflurane and continuous monitoring of breathing, heart rate and temperature was performed.

### Organ and body measurements

Following cardiac function assessment, animals were euthanised by cardiac puncture under anaesthesia of 2% isoflurane, after which blood was drawn, hearts were excised and weighed. Atria, right ventricle (RV) and left ventricle (LV) were separated and snap frozen in liquid nitrogen for molecular analysis.

### Histology

Mid-papillary sections of LV were preserved for histological purposes. Sections were fixated in 4% paraformaldehyde for 24 h, which was followed by dehydration steps and embedding of samples in paraffin (Leica, TP 1020, Germany). For cryopreservation, LV samples were fixated in TissueTec and slowly snap frozen in liquid nitrogen.

#### Cross sectional area

4 μm sections were deparaffinized and stained with 4′,6-diamidino-2-phenylindole (DAPI) (ab H-1200, Vector laboratories, USA) and wheat germ agglutinin-FITC (WGA) (ab L4895, Sigma-Aldrich, USA) to determine cardiomyocyte cross sectional area, as a parameter for cardiomyocyte size^17^. Fluorescent microscopy (Leica, ctr 600, Leica Microsystems, Germany) was performed to obtain images of transverse sections at 20× magnification, which were quantified using Fiji-ImageJ software (Fiji- ImageJ version of Java 6, National Institutes of Health, USA). In total, 30–40 cardiomyocytes were manually selected per animal.

#### Cardiomyocyte length and width

10 μm sections were deparaffinized and stained with DAPI, WGA and an antibody that recognizes Desmin (ab SC-7559 Santa Cruz, USA). Fluorescent microscopy (Leica, ctr 600, Leica Microsystems, Germany) was performed to obtain images of longitudinal 3D Z-stacks at 20× magnification (Supplementary Fig. S2). The cardiomyocyte length and width were measured at the mid-nuclear level of individual cardiomyocytes, using the desmosomes as landmarks using Fiji-ImageJ software (Fiji- ImageJ version of Java 6, National Institutes of Health, USA). In total, 30–40 cardiomyocytes were randomly selected per animal.

#### Fibrosis

4 μm sections were deparaffinized and stained with Masson Trichrome staining to determine the level of interstitial fibrosis present^18^. Images were generated at 40× magnification with Hamamatsu (Hamamatsu Photonics, Japan) and quantified using Aperio ImageScope software (Version 11, Leica Microsystems, Germany).

#### Capillary density

Cryosections were incubated with an antibody against endothelial antigen CD31 to stain for capillaries as a measure of angiogenesis (DIA-310, Dianova, Germany)^19^. Quantification of capillaries was performed by selecting random fields at 40× magnification (Leica, ctr 600, Leica Microsystems, Germany).

#### Electron microscopy

To evaluate the compartmental localization of AKIP1 in the cardiomyocyte, we performed large-scale electron microscopy (EM), known as nanotomy (for nano-anatomy)^20^. Detailed description of the methodology was reported previously^20^. In brief, following sacrifice, LV samples were sliced and fixated immediately in 2% glutaraldehyde and 2% paraformaldehyde solution in 0,1 M sodium cacodylate and postfixed in 1% osmiumtetraoxide and 1.5% potassium ferrocyanide. Samples were dehydrated, embedded in EPON epoxy resin, and sectioned. Ultrathin Sections (80 nm) were contrasted using 4% neodymium acetate. In addition, sections were immunolabelled as described previously^21^. In brief, samples were etched with 1% periodic acid for 10 min, followed by a 30-min blocking step: 1% bovine serum albumin (BSA; Sanquin, the Netherlands) in tris-buffered saline (TBS), pH 7.4. Next, AKIP1 primary antibody (Supplementary Table S2) was incubated for 2 h followed by washing and subsequent incubation for 1 h with biotinylated secondary antibody (goat-anti-rabbit; 1:400, Dako, Denmark), followed by washing steps. Finally, streptavidin conjugated QD655 (1:1000, Life Technologies, United States) was incubated for 1 h. Sections were imaged using scanning and transmission electron microscopy (STEM) (Zeiss Supra55, Oberkochen Germany). Images were processed into a nanotomy ‘map’ using an external scan generator ATLAS5 (Fibics, Canada). TIFF files were then exported to html files available at www.nanotomy.org. To identify elemental composition of structural changes observed on EM images, we performed additional analysis using the energy-dispersive X-ray (EDX) detector X-max (Oxford Instruments, United Kingdom)^20^.

#### Quantitative real-time polymerase chain reaction (qRT-PCR)

To determine alterations in mRNA expression levels after exercise in AKIP1-TG mice, qRT-PCR was performed. Total RNA was isolated from snap frozen LV tissue using Trizol reagent (Invitrogen Corporation, USA), as performed before^19^. Using Nanodrop software (ThermoFisher, USA), RNA concentrations were quantified and tested for purity. Next, equal RNA quantities (500 ng) were reverse transcribed to cDNA with Quantitect Reverse Transcription kit (Qiagen, the Netherlands, no. 205313). cDNA was amplified with qRT-PCR for specific primers, as presented in Supplementary Table S1. Standard running protocol was performed: 3 min at 95 °C, followed by 35 cycles of (1) 15 s at 95 °C, (2) 30 s at 60 °C; followed by a dissociation step and melting steps (Bio-Rad CFX384, USA). Results were analysed using the ddCT method normalizing for housekeeping gene 36b4 and WT Sed control group.

#### Western blot

To assess physiological alterations in AKIP1-TG mice on protein level, we performed Western blot. Total protein was isolated from snap frozen LV tissue using radioimmunoprecipitation assay (RIPA) buffer with phosphatase and protease inhibitors (Sigma-Aldrich, USA), as previously described^22^. Protein concentrations were quantified using BCA protein assay reagent (Pierce. No. 232250, ThermoFisher, USA). Samples with similar proteins concentrations of 10 μg/15 μl, including 5X loading buffer and RIPA buffer, were boiled at 99 °C for five minutes before loading onto sodium dodecyl sulphate–polyacrylamide gel electrophoresis (SDS-PAGE). After finalization of SDS-PAGE, proteins were transferred by semi-dry blotting onto polyvinylidene difluoride (PVDF) membranes. Following blotting and blocking, membranes were incubated with specific primary antibodies at 4 °C overnight: a list of primary antibodies used can be found in Supplementary Table S2. After one hour secondary antibody incubation, proteins were detected in ImageQuant machine with enhanced chemiluminescence solution (Pierce, ThermoFisher, USA). Analysis of images was performed in Fiji-ImageJ software (Fiji- ImageJ version of Java 6, USA) and quantifications were normalized for GAPDH or α-Tubulin as loading controls.

#### Statistical analysis

Data are presented with mean and standard error of the mean (SEM). Statistical analysis of two groups was performed with Student’s *t*-test, if data was normally distributed. If data was not normally distributed, statistical analysis of two groups was performed with Mann–Whitney U test. A *p* value < 0.05 was considered statistically significant. When comparing multiple groups, Two-way ANOVA with post hoc Tukey’s test was performed. Two-way ANOVA was chosen over one-way ANOVA to determine the interactive effects of both genotype and exercise. If the Two-way ANOVA showed a statistical difference (*p* value < 0.05), post hoc Tukey’s test was performed to observe differences for each group^14^. Statistical analysis was performed using GraphPad Prism software (Version 7, USA).

## Results

### Exercise performance in AKIP1-TG mice is not affected

AKIP1 transgenic mice were phenotypically normal, with the exception of a mild reduction in left ventricular systolic blood pressure and left ventricular ejection fraction which we have described before^14^, cardiac structure and function were comparable between sedentary AKIP1-TG and WT groups. Heart rate, body and lung weight were also comparable between sedentary AKIP1-TG and WT mice (Supplementary Table S3). In this study, we will assess several molecular markers associated with exercise-induced cardiac hypertrophy. To ensure that these markers were not altered in sedentary mice, we also performed analysis of these markers in mice that did not perform exercise, which remained unchanged (Supplementary Fig. S3).

Wild type mice ran an average distance of 7.85 ± 0.75 kms, during 4.96 ± 0.31 h per night, at an average speed of 1.53 ± 0.07 km per hour. All these indices of running performance were comparable between AKIP1-TG and WT mice, indicating that both exercise capacity and workload during the study were comparable (Fig. 1A–F).

### Physiological cardiac hypertrophy is increased in AKIP1-TG mice, but initial cardiomyocyte size analysis depicts no differences

**Figure 2:**
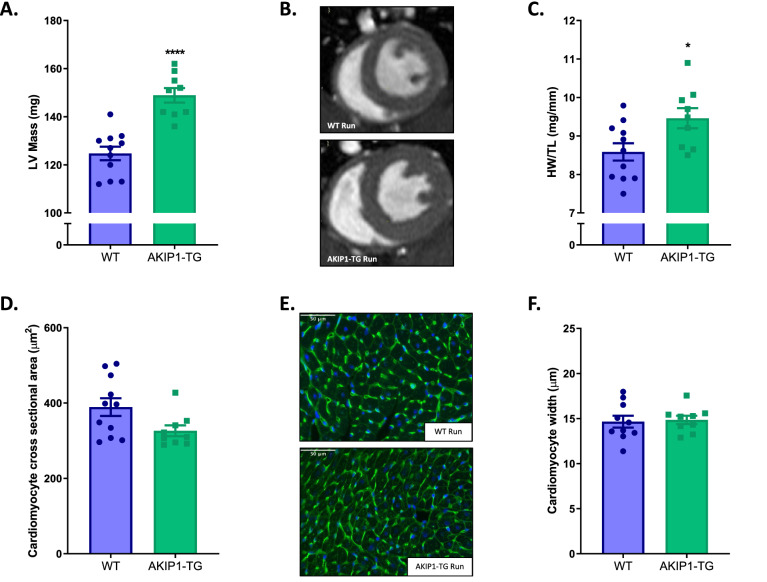
Cardiac hypertrophy parameters and cardiomyocyte size after voluntary wheel running in both WT and AKIP1-TG mice. Shown are, the first three panels as indicators for cardiac hypertrophy, (N = 9–11/group), in (**A**) left ventricular mass (LV Mass) detected from MRI shown in milligrams (mg) with its typical examples in panel (**B**) and in (**C**) the heart weight to tibia length ratio (HW/TL) is shown in milligrams per millimetres (mg/mm). In panel (**D**), cardiomyocyte cross sectional area in micrometres squared (μm^2^) is shown, (N = 9–11/group), (**E**) shows typical examples for cardiomyocyte cross sectional area, scale bars indicate 50 μm. In panel (**F**)*,* cardiomyocyte width in micrometres (μm), (N = 9–10/group), the latter three panels present initial cardiomyocyte size analysis. Mice included were aged 8–12 weeks old. WT = wild type mice, AKIP1-TG = AKIP1 transgenic mice, Run = Running. Graphs represent mean ± standard error of the mean (SEM). Statistical analysis was performed with Student’s *t-*test or Mann–Whitney U test, < 0.05 was considered statistically significant. WT Run *vs.* TG Run: p* < 0.05, p**** < 0.0001.

We next assessed the effect of AKIP1 on exercise-induced changes in cardiac structure and function using cardiac MRI and pressure–volume measurements after four weeks of voluntary wheel running. Cardiac volumes and left ventricular ejection fraction determined with MRI were comparable between AKIP1-TG and WT mice after exercise (Supplementary Table S4). Similarly, cardiac pressures were also comparable between groups upon exercise, with again the exception for a mild reduction in LV systolic blood pressure (Supplementary Table S4). Exercise-induced increases in LV mass on MRI, were, however, markedly increased in AKIP1-TG compared to WT mice (Fig. 2A,B). These increases in LV-mass detected with MRI were paralleled by a similar increase in heart weight to tibia length ratio (Fig. 2C).

To determine whether the increase in heart weight corresponded with an increase in cardiomyocyte size, cardiomyocyte cross sectional area was determined by performing a wheat germ agglutinin staining. Surprisingly, cardiomyocyte cross sectional area was similar after exercise in AKIP1-TG and WT mice (Fig. 2D,E). In accordance with cardiomyocyte cross sectional area, cardiomyocyte width did not differ between the running groups (Fig. 2F).

### AKIP1-induced cardiac hypertrophy is dependent upon cardiomyocyte elongation mediated by dephosphorylation of serum response factor

**Figure 3:**
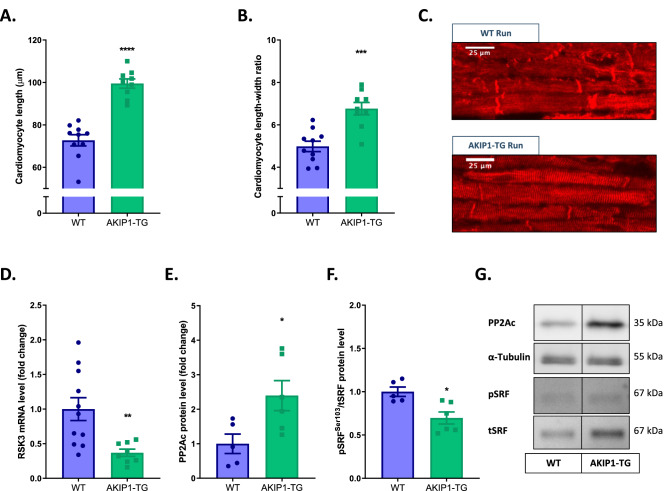
Cardiomyocyte length analysis and its regulation after voluntary wheel running in both WT and AKIP1-TG mice. Shown are (**A**) cardiomyocyte length in micrometres (μm), (N = 9–10/group), (**B**) cardiomyocyte length to width ratio (N = 9–10/group), and panel (**C**) represents typical examples for cardiomyocyte length and width measurements, scale bars indicate 25 μm. In panel (**D**) mRNA expression level of RSK3 is shown, (N = 8–11/group). Protein level of PP2Ac normalized for α-Tubulin is presented in panel (**E**)*,* phosphorylation to total SRF (pSRF^Ser103^/tSRF) in panel (**F**) and representative images of Western blots are presented in panel (**G**), (N = 5–6/group). Representative Western blot images are taken from the same blot, but not always contiguous; full and original Western blots are included in Supplementary Figures. Mice included were aged 8–12 weeks old. WT = wild type mice, AKIP1-TG = AKIP1 transgenic mice, Run = Running, RSK3 = p90 ribosomal S6 kinase 3, PP2Ac = protein phosphatase 2A catalytic subunit, α-Tubulin = alpha-tubulin, SRF = serum response factor. Graphs represent mean ± standard error of the mean (SEM). Statistical analysis was performed with Student’s *t-*test or Mann–Whitney U test, < 0.05 was considered statistically significant. WT Run *vs.* TG Run: p* < 0.05, p** < 0.01, p*** < 0.001, p**** < 0.0001.

As adaptive cardiomyocyte growth in response to endurance exercise is predominantly eccentric, we hypothesized that the increase in cardiac mass in AKIP1-TG mice could be explained by cardiomyocyte elongation^1,23^. To test this hypothesis, we analysed cardiomyocyte length to width ratio using 3D Z-stacks on myocardial sections stained with DAPI, WGA and Desmin to identify cardiomyocyte borders at the desmosome (Supplementary Fig. S2). Intriguingly, cardiomyocyte length was significantly increased in response to exercise in AKIP1-TG mice compared to WT mice (WT Run 72.72 ± 2.66 μm *vs.* AKIP1-TG Run 99.48 ± 2.18 μm, *p* < 0.0001), resulting in a significant increase in the cardiomyocyte length to width ratio (Fig. 3A–C). These findings suggest that the exercise-induced increase in cardiac mass observed in AKIP1-TG mice is predominantly explained by elongation of cardiomyocytes.

It has recently been shown that dephosphorylation of serum response factor (SRF) is a prerequisite for eccentric growth of cardiomyocytes. Dephosphorylation of SRF in this context is regulated by downregulation of p90 ribosomal S6 kinase 3 (RSK3) and upregulation of protein phosphatase 2A catalytic subunit (PP2Ac)^24,25^. In support of this mechanism, we observed a significant reduction in mRNA levels of RSK3 and a significant increase of protein expression of PP2Ac, accompanied by dephosphorylation of SRF in AKIP1-TG mice compared to WT mice after exercise (Fig. 3D–G).

### Exercise-induced cardiac hypertrophy in AKIP1-TG mice was associated with physiological angiogenesis and unaltered fibrosis

**Figure 4:**
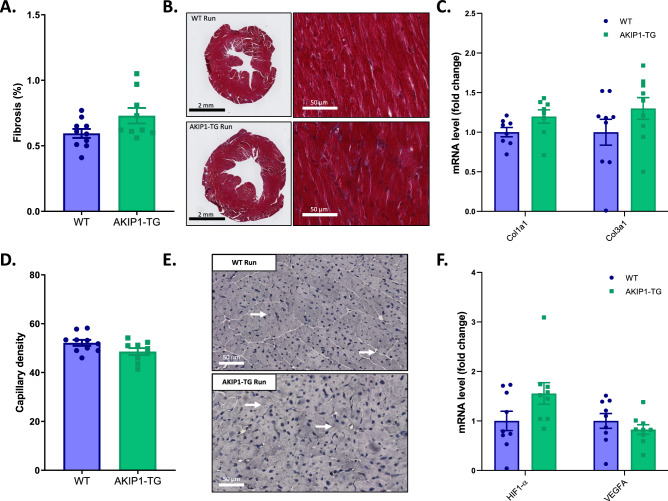
Fibrosis and angiogenesis levels to determine the presence of additional aspects of cardiac hypertrophy. Shown are, (**A**) fibrosis levels from Masson staining (N = 9–10/group) with its typical examples in (**B**)*,* in which scale bars indicate 2 mm in the left panels and 50 μm in the right panels. mRNA levels of collagens are shown in panel (**C**) (N = 8–9/group). In panel (**D**) and (**E**), capillary density (N = 9–10/group) and typical examples are shown, with scale bars indicating 50 μm. Panel (**F**) shows mRNA levels of markers for angiogenesis (N = 9/group). Mice included were aged 8–12 weeks old. WT = wild type mice, AKIP1-TG = AKIP1 transgenic mice, Run = Running, Col1a1 = collagen type 1 alpha 1 chain, Col3a1 = collagen type 3 alpha 1 chain, HIF1-α = hypoxia inducible factor 1 alpha, VEGFA = vascular endothelial growth factor A. Graphs represent mean ± standard error of the mean (SEM). Statistical analysis was performed with Student’s *t-*test or Mann–Whitney U test, < 0.05 was considered statistically significant.

We next aimed to corroborate whether the augmented exercise-induced cardiac hypertrophy in AKIP1-TG mice was adaptive in nature. An important distinction between adaptive and maladaptive hypertrophy is the reduction in capillary density and the development of cardiac fibrosis in the latter condition^1–3^. Interstitial fibrosis detected with Masson Trichrome staining did not differ between WT and AKIP1-TG running mice (Fig. 4A,B). Cardiac expression of common molecular markers of fibrosis, collagens 1 and 3 (Col1a1 and Col3a1 respectively), were also comparable between groups (Fig. 4C). Similarly, capillary density detected with CD31 staining and the expression of angiogenic markers hypoxia-inducible factor 1-alpha (HIF1-α) and vascular endothelial growth factor A (VEGFA) were similar between AKIP1-TG-Run and WT Run mice (Fig. 4C–F). Together, these data suggest that the marked increase in cardiac hypertrophy observed in AKIP1-TG mice is adaptive in nature (Fig. 4A–F).

### AKIP1 localises in clusters in the cardiomyocyte nucleus

**Figure 5:**
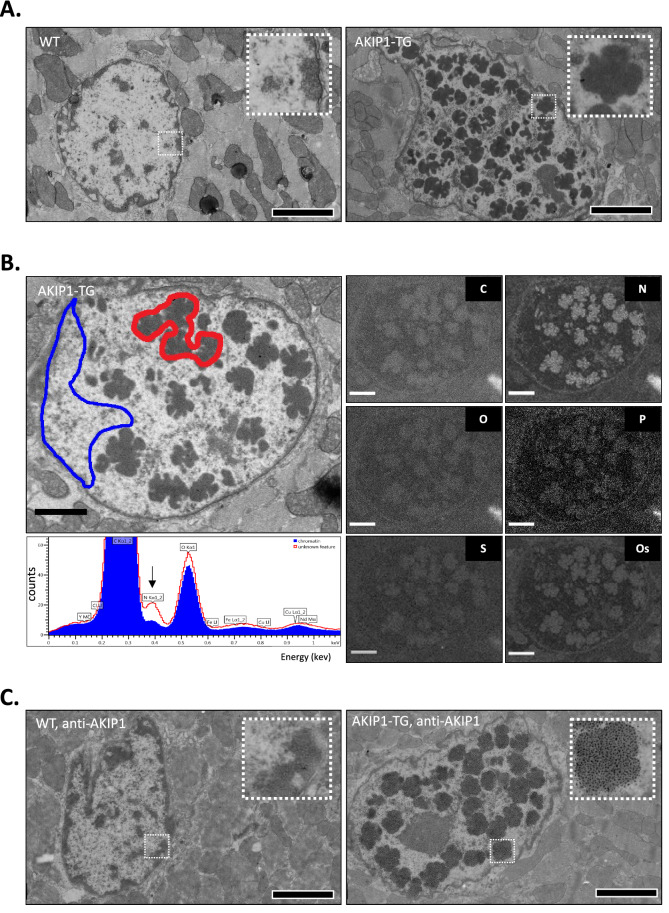
AKIP1 mega clusters localise in the cardiomyocyte nucleus of AKIP1-TG mice. Electron microscopy (EM) mapping to identify various cellular compartments in baseline WT and AKIP1-TG mice. Shown are, in (**A**) a selection of a nucleus and surrounding cytoplasm of WT (left) and AKIP1-TG (right) mice. Note the atypical clusters inside the nucleus. In (**B**) electron image and EDX maps of elements as indicated to identify content of nucleus structures observed in AKIP1-TG mice. Note that the aggregates (red ROI and graph) are enriched in nitrogen (arrow) compared to heterochromatin (blued ROI and graph). (**C**) EM ultra-sections were labelled with anti-AKIP1 antibody. Raw data with zoomable files at high resolution are accessible at www.nanotomy.org. All scale bars indicate 2.5 μm. Mice included were aged 8–12 weeks old. WT = wild type mice, AKIP1-TG = AKIP1 transgenic mice, C = carbon, N = nitrogen, O = oxygen, P = phosphorus, S = sulphur, Os = osmium.

We then determined the localisation of AKIP1 in the cardiomyocyte, which could potentially explain a switch in transcription factors underlying the exercise-induced cardiac hypertrophy. Therefore, we performed electron microscopy (EM) imaging of WT and AKIP1-TG mice. First, we qualitatively assessed EM images and interestingly, we observed structural changes in the nuclear compartments of AKIP1-TG mice (Fig. 5A). To address whether the content of the structural changes in the nuclei did have the fingerprint of protein, DNA or lipid clusters, we performed elemental composition with EDX mapping on the EM scans. These EDX data maps revealed that the structures are rich in nitrogen but not in phosphorus, hinting to protein clusters (Fig. 5B). To confirm whether these protein clusters contain AKIP1, we subsequently performed EM labelling with AKIP1 antibody. Indeed, presence of AKIP1 was observed in the dense nuclear areas in EM scans of AKIP1-TG mice and in WT mice this nuclear labelling was much less, as expected (Fig. 5C). These data suggest that AKIP1 localizes in clusters in the nucleus, potentially aiding in signalosome formation that predisposes for a switch in transcription in response to exercise.

### AKIP1 activates the physiological Akt-C/EBPβ-CITED4 growth pathway upon exercise

Next, we sought to determine the switch in molecular mechanisms underlying AKIP1-induced physiological cardiac hypertrophy, focused on the Akt-C/EBPβ-CITED4 pathway*.* Previous studies suggest that AKIP1 regulates protein kinase B (Akt) in association with physiological cardiac hypertrophy^11,13^. Metacore transcription factor analysis also suggested a transcriptional regulation of CCAAT Enhancer Binding Protein Beta (C/EBPβ) by AKIP1 in sedentary mice. C/EBPβ is a transcription factor that has been established as a negative regulator of exercise-induced physiological growth^26,27^. Specifically, C/EBPβ is downregulated by phosphorylated protein kinase B (Akt), resulting in the de-repression of downstream genes that promote physiological growth^26,27^. Simultaneously, reduction of C/EBPβ induces de-repression of Cbp/p300 interacting transactivator with Glu/Asp rich carboxy-terminal domain 4 (CITED4)^26–28^. CITED4 has recently emerged as a nodal point in the physiological adaptation to both physiological and pathological cardiac stress and intriguingly also regulates cardiomyocyte elongation^29–31^. Mechanistically, CITED4 controls the regulation of mTOR signalling through the inhibition of the DNA-damage-inducible transcript 4-like (DdiT4L)^30–33^.

**Figure 6:**
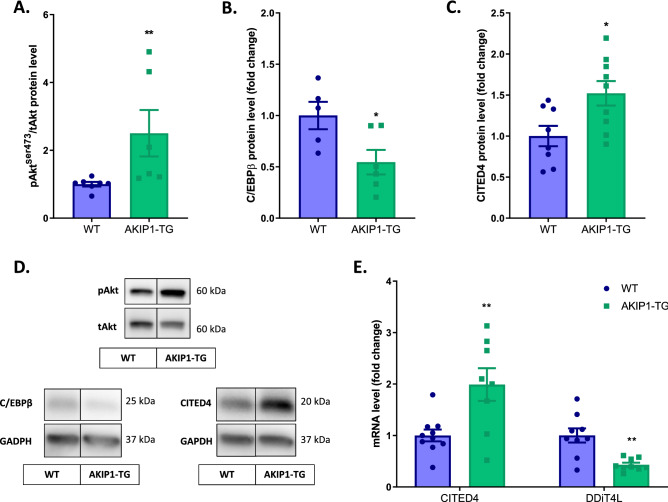
Molecular analysis of the physiological growth pathway: Akt-C/EBPß-CITED4 activation. mRNA and protein level analysis of the exercise-induced physiological growth pathway in AKIP1-TG mice. Shown are, (**A**) ratio of phosphorylated Akt to total Akt (pAkt^Ser473^/tAkt) at protein level, (N = 6–7/group), (**B**) C/EBPß protein levels normalized for GAPDH, (N = 5–6/group), (**C**) CITED4 protein levels normalized for GAPDH, (N = 8–9/group), (**D**) typical examples of the described Western blots and (**E**) CITED4 and DDiT4L mRNA levels, normalized for housekeeping gene 36B4, (N = 8–10/group). Representative Western blot images are taken from the same blot, but not always contiguous; full and original Western blots are included in Supplementary Figures. Mice included were aged 8–12 weeks old. WT = wild type mice, AKIP1-TG = AKIP1 transgenic mice, Run = Running, Akt = protein kinase B, C/EBPß = CCAAT Enhancer Binding Protein Beta, CITED4 = Cbp/p300 interacting transactivator with Glu/Asp rich carboxy-terminal domain 4, DDiT4L = DNA-damage-inducible transcript 4-like. Graphs represent mean ± standard error of the mean (SEM). Statistical analysis was performed with Student’s *t-*test or Mann–Whitney U test, < 0.05 was considered statistically significant. WT Run *vs.* TG Run: p* < 0.05, p** < 0.01.

**Figure 7:**
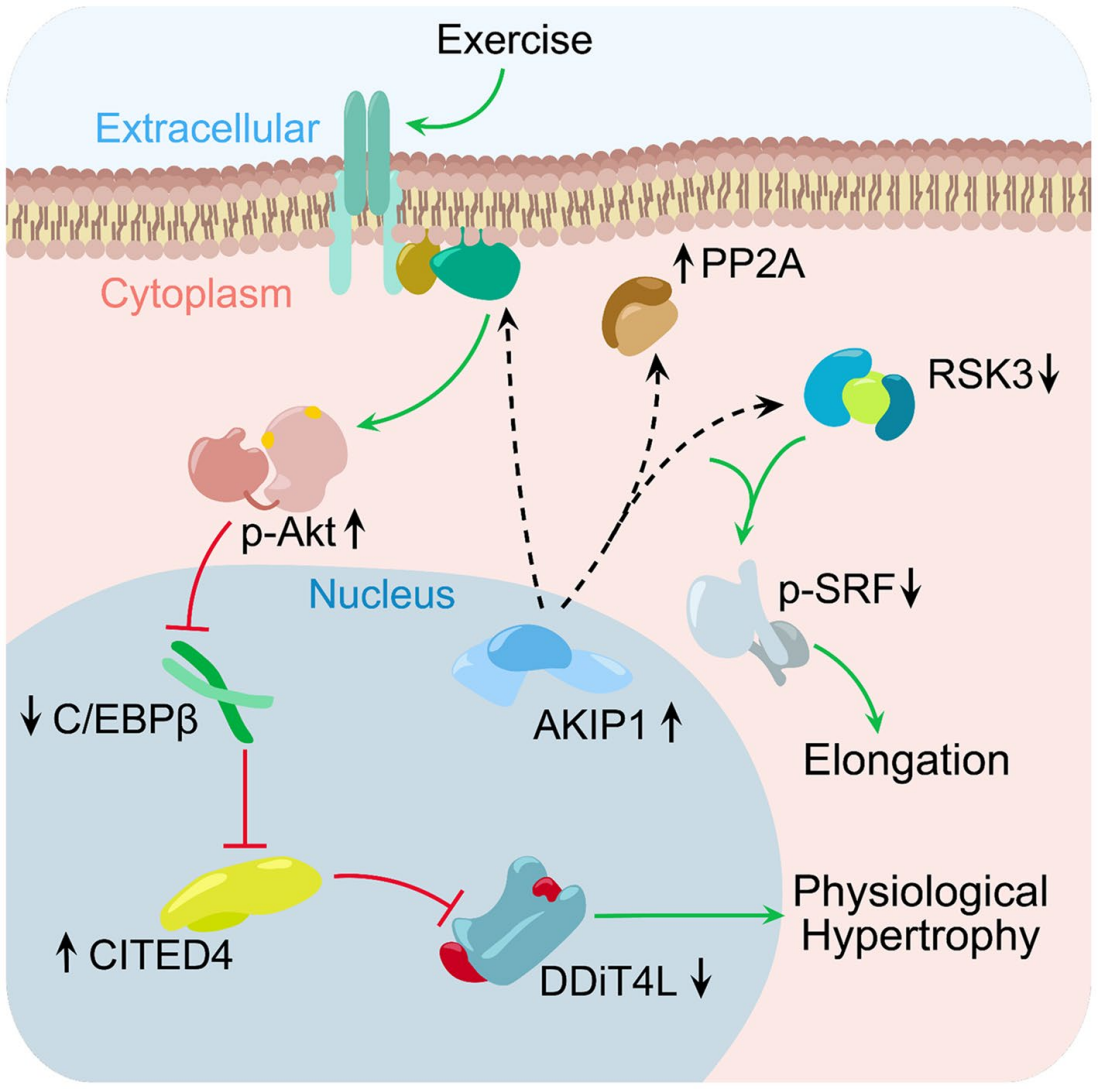
Graphical abstract depicting the molecular signal transduction pathways associated with elongation-mediated physiological cardiac hypertrophy. Increased levels of AKIP1 in combination with an exercise regimen induces activation of the RSK3-PP2Ac-SRF and Akt-C/EBPβ-CITED4 pathway. It remains unknown whether AKIP1 exerts direct or indirect effects, nonetheless increased AKIP1 in cardiomyocytes activates phosphorylation of Akt, which in turn de-represses C/EBPß. The C/EBPß downstream factor CITED4, is subsequently upregulated which results in inhibition of DDiT4L. Consequentially, activation of this pathway in turn leads to established physiological cardiac hypertrophy. Simultaneously, AKIP1 overexpression in concordance with exercise reduces levels of RSK3 and enhances levels of PP2Ac. This synchronized programming dephosphorylates SRF, which entails a molecular switch towards physiological elongation of cardiomyocytes. AKIP1 = A Kinase Interacting Protein 1, Akt = protein kinase B, C/EBPß = CCAAT Enhancer Binding Protein Beta, CITED4 = Cbp/p300 interacting transactivator with Glu/Asp rich carboxy-terminal domain 4, DDiT4L = DNA-damage-inducible transcript 4-like, RSK3 = p90 ribosomal S6 kinase 3, PP2Ac = protein phosphatase 2A catalytic subunit, SRF = serum response factor.

We next determined whether the AKIP1-induced eccentric cardiac hypertrophy was in fact associated with activation of this pivotal exercise-associated Akt-C/EBPβ-CITED4 growth signalling pathway. As expected, phosphorylation of Akt after exercise was significantly increased in AKIP1-TG mice compared to WT mice (Fig. 6A). Furthermore, C/EBPβ protein expression was significantly reduced in AKIP1-TG mice (Fig. 6B) and CITED4 mRNA and protein levels were significantly increased in AKIP1-TG mice compared to WT mice after exercise (Fig. 6C–E). Finally, we also assessed the potential downstream factor of C/EBPβ and CITED4, DdiT4L, which is known to suppress the mTOR activity. In corroboration with our previous findings, we observed a significant inhibition of DdiT4L in AKIP1-TG mice after exercise (Fig. 6E). The data are summarized in Fig. 7.

## Discussion

To define the contribution of AKIP1 to physiological cardiac hypertrophy, we compared the cardiac response to four weeks of voluntary wheel running exercise between AKIP1-TG and WT mice. While cardiac structure and function were comparable between sedentary AKIP1-TG and WT mice, exercise-induced cardiac hypertrophy was substantially enhanced by AKIP1. Intriguingly, the increase in cardiac mass was predominantly associated with cardiomyocyte elongation. Accordingly, with molecular analysis we observed that AKIP1 reduced mRNA expression of RSK3, resulting in PP2Ac-mediated dephosphorylation of SRF, consistent with increased signalling through the PP2Ac-SRF signalosome. Subsequently, we determined that AKIP1 localizes in clusters in the cardiomyocyte nucleus, causing a potential predisposition for signalosome formation to stimulate a switch in transcription upon exercise. Furthermore, we observed mechanistic changes involving that AKIP1 promoted exercise-induced phosphorylation of Akt, downregulation of C/EBPβ and subsequent de-repression of CITED4. The changes in C/EBPβ and CITED4 expression was accompanied by canonical changes in their downstream target DdiT4L. In conclusion, our studies revealed that the signalling adaptor AKIP1 is a novel regulator of cardiomyocyte elongation and physiological cardiac hypertrophy, which is associated with the activation of two growth signalling pathways (summarized in Fig. 7). AKIP1 may therefore serve as a nodal point for cardiomyocyte elongation and physiological reprogramming of cardiac muscle.

AKIP1 was first discovered as a breast cancer associated gene (BCA)^34^, but was soon also found in several normal tissues, including the heart^9^. Myocardial expression of AKIP1 is increased in physiological hypertrophy^10,11^ and overexpression of AKIP1 induces hypertrophy in cultured cardiomyocytes that resembles physiological cardiac hypertrophy in vivo^12,13^. Furthermore, AKIP1 did not influence cardiac remodelling in response to various forms of pathologic cardiac stress, indicating that its role in the pathological setting is limited^14^. Our discovery that AKIP1 stimulates physiological cardiac growth in vivo, by activating two distinct but complementary physiological signalling pathways, provides strong evidence that AKIP1 is a bona fide regulator of physiological cardiac hypertrophy.

While cardiac hypertrophy can be classified as physiological or pathological depending on the underlying stimulus and the associated loading conditions, it can also be subdivided as concentric or eccentric depending on ventricular geometry^1,2^. Concentric hypertrophy is characterized by asymmetrical growth of cardiomyocytes in width, whereas eccentric hypertrophy is characterized by preferential myocyte elongation^1,2^. Endurance exercise has mostly been associated with eccentric hypertrophy and our finding that AKIP1 promotes cardiomyocyte elongation fits this paradigm^1,4,35,36^. The mechanisms responsible for these distinct growth patterns were recently found to be governed by a mAKAPβ-SRF signalosome, which allows close interaction between SRF and its co-factors, RSK3 and PP2Ac^24,25^. Our finding that AKIP1 promotes dephosphorylation of SRF, associated with repression of RSK3 and upregulation of PP2Ac, confirms that AKIP1 promotes cardiomyocyte elongation by interacting with this signalosome. Of note, in our study, cardiomyocyte elongation is not associated with changes in cardiac function and cardiac dilatation. In fact, left ventricular end diastolic volume (LVEDV) was numerically lower in AKIP1-TG mice after exercise, suggesting that the observed eccentric cardiac hypertrophy is adaptive in nature.

Similar to what has been observed in cultured cardiomyocytes, AKIP1 promoted Akt phosphorylation after exercise, and this study provides additional evidence for association with canonical activation of the downstream C/EBPβ-CITED4-DdiT4L pathway^13^. This exercise-induced physiological growth pathway has recently been discovered to govern beneficial adaptation to various forms of physiological and pathological stress^26–31,37^. Firstly, this pathway is associated with generation of physiological growth and is associated with beneficial cardiac remodelling^26–31,37^. Secondly, CITED4 has been found to regulate cardiomyocyte elongation^31^, suggesting that the cardiomyocyte elongation observed in our study may be explained by the coordinated dephosphorylation of SRF and activation of CITED4. The fact that AKIP1 activates two distinct pathways responsible for cardiomyocyte elongation and remodelling, strongly suggests that AKIP1 represents a nodal point in the regulation of cardiomyocyte reprogramming.

Regulation of Akt activity is a multifactorial process, that involves many direct as well as indirect interactions^1,6^. As AKIP1 has been identified as a signalling adaptor protein, it is possible that AKIP1 directly interacts with Akt, its activators or its phosphatases. Recent evidence suggests that microRNA-222 (miR-222) regulates the exercise-induced Akt-C/EBPβ-CITED4 pathway^28,38^. Potentially, this could also involve interaction with AKIP1. Also, miR-223 has been associated with physiological cardiac hypertrophy, in a study in which AKIP1 levels were also determined to be upregulated^11^. Hence, further investigation into whether AKIP1 modulates the expression of miR’s including miR-222 and miR-223, would potentially offer further insight into AKIP1 as a therapeutic target focused on activating cardiac remodelling. An additional mechanistic question is whether the RSK3-PP2Ac-SRF and Akt-C/EBPβ-CITED4 pathways are interrelated. Intriguingly, a link between the two pathways has been described previously, involving a direct interaction between SRF and C/EBPβ^27^. Therefore, these pathways may indeed be complementary, resulting in dual elongation pathways for physiological cardiac remodelling, a possibility that requires future study.

The functional role of AKIP1 may expand beyond its role in elongating cardiomyocytes upon exercise training. In particular, AKIP1 may also regulate mitochondrial adaptations integral to the physiological cardiac growth response. We previously showed that overexpression of AKIP1 in cultured cardiomyocytes improves mitochondrial respiration, accompanied by enhanced ATP production and reductions in mitochondrial ROS^12^. AKIP1 also appears to localize to mitochondria^14^, and two independent studies demonstrated that AKIP1 protected against myocardial ischemia/reperfusion injury, at least in part by mitigating mitochondrial cell death pathways^14,39^. These studies suggest that AKIP1 could influence mitochondrial adaptations to physiological stress, resulting in a contribution to physiological hypertrophy. To therefore determine whether AKIP1 can indeed serve as a therapeutic target and regulate physiological cardiac hypertrophy, future studies should focus on the role of AKIP1 on mitochondrial function.

### Strengths and limitations

A major strength of our analysis is the detailed myocardial phenotyping using cardiac MRI, pressure volume loop analysis as well as state of the art histological and molecular analysis. The primary limitation of our study is the fact that we employed voluntary exercise. Although this represents a well-established method to study physiological hypertrophy, the total workload is typically lower than observed with forced exercise and many factors can potentially influence the amount of exercise performed^15,40^. In our study exercise performance and total workload were similar for both groups, allowing a fair comparison of the hypertrophic response. Nevertheless, exercise-induced hypertrophy was very subtle in wild type mice and the results may have been different if forced exercise would have been employed. In addition, we used an animal model with marked overexpression of the protein of interest and we therefore cannot exclude off-target effects or the ability to fully recapitulate the human situation. The development of a cardiomyocyte specific knock-out mouse model would aid in bypassing this limitation.

### Future perspectives

We have provided evidence that AKIP1 activates the RSK3-PP2Ac-SRF and Akt-C/EBPβ-CITED4 pathway in response to exercise accompanied by enhanced physiological hypertrophy. However, to further explore and potentially pursue towards development of therapeutic therapies, it is of essence to further understand *how* AKIP1 activates these pathways. The role of AKIP1 may be mediated through factors upstream of Akt such as the IGF1 receptor or PI3K, but it may also interact directly with Akt or its phosphatases. Of note, the Akt phosphatase PP2Ac was found to be upregulated in our study. Alternatively, AKIP1 may act upon the direct interaction between these two pathways, possibly at the C/EBPβ-SRF link. Additionally, it should be further validated whether changes in these signalling pathways are causing physiological cardiac hypertrophy, or whether these changes are resulting from physiological cardiac hypertrophy. Future research attempts involving in vitro and knock-out mouse models should provide more in-depth mechanistic insights into the mechanisms responsible for AKIP1-mediated activation of these signalling pathways.

## Conclusion

Taken together, we provide the evidence that AKIP1 promotes cardiomyocyte elongation and physiological cardiac remodelling with activation of the RSK3-PP2Ac-SRF and Akt-C/EBPβ-CITED4 pathway. This study provides evidence that AKIP1 may be a potential therapeutic target to shift pathological remodelling towards a more physiological phenotype.

## Supplementary Information

Supplementary Information.

## Data Availability

The data from the current study are available from the corresponding author on reasonable request.
