# Supplementary material for: A Kinase Interacting Protein 1 (AKIP1) promotes cardiomyocyte elongation and physiological cardiac remodelling
Source: Sci Rep. 2023 Mar 10;13:4046. doi: 10.1038/s41598-023-30514-1 (PMC10006410; doi:10.1038/s41598-023-30514-1)
Supplement: Supplementary file 1 — Supplementary Information. [file 41598_2023_30514_MOESM1_ESM.pdf]

Supplementary information

A Kinase Interacting Protein 1 (AKIP1) promotes cardiomyocyte elongation and physiological cardiac remodelling

Kirsten T. Nijholt, BSc[1], Pablo I. Sánchez-Aguilera, MSc[1], Harmen G. Booij MD, PhD[1], Silke U. Oberdorf-Maass, BSc[1], Martin M. Dokter, BSc[1], Anouk H.G. Wolters, BSc[2], Ben N.G. Giepmans, PhD[2], Wiek H. van Gilst, PhD[1], Joan H. Brown, PhD[3], Rudolf A. de Boer, MD, PhD[1], Herman H.W. Silljé, PhD[1], B. Daan Westenbrink, MD, PhD[1]

[1]Department of Cardiology, University Medical Centre Groningen, University of Groningen, Groningen, The Netherlands

[2]Department of Biomedical Sciences of Cells and Systems, University Medical Centre Groningen, University of Groningen, Groningen, The Netherlands

[3]Department of Pharmacology, University of California San Diego, La Jolla, United States of America

Corresponding author:
B.D. Westenbrink, MD, PhD
Department of Cardiology, University Medical Centre Groningen
Hanzeplein 1, 9713 GZ Groningen, The Netherlands
P.O. Box 30.001, 9700 RB Groningen, The Netherlands
Phone: +31 50 361 23 55, fax: +31 50 361 13 47, email: b.d.westenbrink@umcg.nl

Short title: AKIP1 promotes physiological cardiac hypertrophy

Tables

Table S1. Primer sequences.

	**5'-3' Forward**	**5'-3' Reverse**
36B4	AAGCGCGTCCTGGCATTGTC	GCAGCCGCAAATGCAGATGG
AKIP1	AGAGGCGAGACATCTGTTGG	TAGGTCCCTGGGTAGACTTC
RSK3	CTCAGCAACGCCTAACAGCAG	CCATGGCACCCTTCACTAGATG
PP2Ac	GGCATCATGGACGAGAAGTTG	TTCTCGCAGAGGCTCTTGAC
Col1a1	AGAGCATGACCGATGGATTC	CGCTGTTCTTGCAGTGATAG
Col3a1	ATATGCCCACAGCCTTCTAC	CCACCAGTTGGACATGATTC
HIF-1α	CACAGAAATGGCCCAGTGAG	CCTTCCACGTTGCTGACTTG
VEGFA	ACTGGACCCTGGCTTTACTG	CAGTAGCTTCGCTGGTAGAC
CITED4	TGACAGTTGGGTCTCGCTTC	TCCGAAGGCTGGTTCAGTTC
DDiT4L	CTGTCCAAGTCAAAGCAAACC	CATAACACAGCCCCGAAGG

Table S1. Primer sequences for the following genes: *(1)* 36B4, housekeeping gene; *(2)* AKIP1, a kinase interacting protein 1; *(3)* RSK3, p90 ribosomal S6 kinase 3; *(4)* PP2Ac, protein phosphatase 2A catalytic subunit; *(5)* Col1a1, collagen type 1 alpha 1 chain; *(6)* Col3a1, collagen type 3 alpha 1 chain; *(7)* HIF-1α, hypoxia inducible factor 1 alpha; *(8)* VEGFA, vascular endothelial growth factor A; *(9)* CITED4, Cbp/p300 interacting transactivator with Glu/Asp rich carboxy-terminal domain 4; *(10)* DDIT4l, DNA damage-inducible transcript 4-like protein.

Table S2. Antibody list.

	Primary antibody
α-Tubulin	T5168, Sigma-Aldrich, USA
GAPDH	10R-G109A, Fitzgerald, USA
AKIP1	Custom made at our lab as described previously[1]
$pSRF^{Ser103}$	4261S, Cell Signalling, UK
tSRF	5147T, Cell Signalling, UK
PP2Ac	PA5-17510, Thermo Fisher, USA
$pAkt^{Ser473}$	4060, Cell Signalling, UK
tAkt	4691, Cell Signalling, UK
C/EBPβ	Ab32358, Abcam, UK
CITED4	AV37255, Sigma-Aldrich, USA

Table S2. Antibodies used for the following proteins: *(1)* α-Tubulin, alpha tubulin; *(2)* GAPDH, glyceraldehyde 3-phosphate dehydrogenase; *(3)* AKIP1, a kinase interacting protein 1; *(4)* pSRF, phosphorylated serum response factor; *(5)* tSRF, total serum response factor; *(6)* PP2Ac, protein phosphatase 2A catalytic subunit; *(7)* $pAkt^{Ser473}$, phosphorylated protein kinase B at serine site 473; *(8)* tAkt, total protein kinase B; *(9)* C/EBPβ, CCAAT enhancer binding protein beta; *(10)* CITED4, Cbp/p300 interacting transactivator with Glu/Asp rich carboxy-terminal domain 4.

Table S3. Body- and lung weight and cardiac parameters.

	WT Sed (N=9-10)	AKIP1-TG Sed (N=5-6)
Body weight (g)	27.78 ± 0.47	27.63 ± 0.39
Lung weight (mg)	157.5 ± 7.5	160.2 ± 5.5
Heart rate (bpm)	465 ± 15	471 ± 18
LVEDP (mmHg)	11.16 ± 0.97	11.87 ± 1.94
LVESP (mmHg)	102.02 ± 0.80	96.87 ± 0.60***
dPdtmax/Pmax (s^{-1})	74.44 ± 3.33	67.31 ± 0.94
dPdtmin/Pmax (s^{-1})	-72.29 ± 3.77	-60.90 ± 1.86*
Tau/cycle length (ms/s)	59.11 ± 1.91	67.06 ± 3.93
LVEDV (μl)	56.84 ± 1.53	61.53 ± 2.91
LVEF (%)	58.51 ± 1.31	51.66 ± 1.18*
LV Mass (mg)	92.89 ± 2.87	101.6 ± 4.77
HW/TL (mg/mm)	8.41 ± 0.12	8.76 ± 0.12

Table S3. Body weight (BW) after experimental period in grams (g); lung weight in milligrams (mg); heart rate (HR) in beats per minute (bpm); left ventricular end diastolic and systolic pressure (LVEDP and LVESP) in millimetre of mercury (mmHg); maximal rate of rise and fall of left ventricular pressure corrected for afterload (dPdtmax/Pmax and dPdtmin/Pmax); Tau corrected for cycle length (Tau/cycle length) in milliseconds per seconds (ms/s); left ventricular end diastolic volume (LVEDV) in microliters (μl) on MRI; left ventricular ejection fraction (LVEF) in percentages (%) on MRI; left ventricular (LV) mass on MRI in milligrams (mg); heart weight normalized for tibia length (HW/TL) in milligrams per millimetre (mg/mm). N= 5-10 mice/per group. WT= wild type mice, AKIP1-TG= AKIP1 transgenic mice, Sed= Sedentary.Values represent mean ± standard error of the mean (SEM). Statistical analysis was performed with Student's *t*-test or Mann-Whitney U test, <0.05 was considered statistically significant. WT Sed *vs.* TG Sed: p*<0.05. p***<0.001.

Table S4. Body- and lung weight and cardiac parameters.

	WT Run (N=11)	AKIP1-TG Run (N=9)
Body weight (g)	27.42 ± 0.56	27.72 ± 0.36
Lung weight (mg)	170.4 ± 4.49	159.7 ± 2.7
Heart rate (bpm)	453 ± 15	425 ± 14
LVEDP (mmHg)	12.63 ± 1.31	11.37 ± 1.02
LVESP (mmHg)	101.30 ± 2.13	94.58 ± 2.20*
dPdtmax/Pmax (s^{-1})	66.08 ± 2.76	67.93 ± 1.05
dPdtmin/Pmax (s^{-1})	-66.54 ± 2.82	-62.38 ± 1.70
Tau/cycle length (ms/s)	65.20 ± 3.03	60.76 ± 2.31
LVEDV (μl)	61.34 ± 1.48	60.40 ± 2.28
LVEF (%)	57.59 ± 1.16	54.59 ± 2.13
LV Mass (mg)	124.7 ± 2.8	148.9 ± 3.00****
HW/TL (mg/mm)	8.59 ± 0.22	9.46 ± 0.26*

Table S4. Body weight (BW) in grams (g) after four weeks of voluntary wheel running; lung weight in milligrams (mg); heart rate (HR) in beats per minute (bpm); left ventricular end diastolic and systolic pressure (LVEDP and LVESP) in millimetre of mercury (mmHg); maximal rate of rise and fall of left ventricular pressure corrected for afterload (dPdtmax/Pmax and dPdtmin/Pmax); Tau corrected for cycle length (Tau/cycle length) in milliseconds per seconds (ms/s); left ventricular end diastolic volume (LVEDV) in microliters (μl) on MRI; left ventricular ejection fraction (LVEF) in percentages (%) on MRI; left ventricular (LV) mass on MRI in milligrams (mg); heart weight normalized for tibia length (HW/TL) in milligrams per millimetre (mg/mm). N= 9-11 mice/per group. WT= wild type mice, AKIP1-TG= AKIP1 transgenic mice, Run= Running. Values represent mean ± standard error of the mean (SEM). Statistical analysis was performed with Student's *t*-test or Mann-Whitney U test, <0.05 was considered statistically significant. WT Run *vs.* TG Run: p*<0.05, p****<0.0001.

Figures

Supplementary Figure S1. AKIP1 mRNA and protein expression in WT and AKIP1-TG mice.

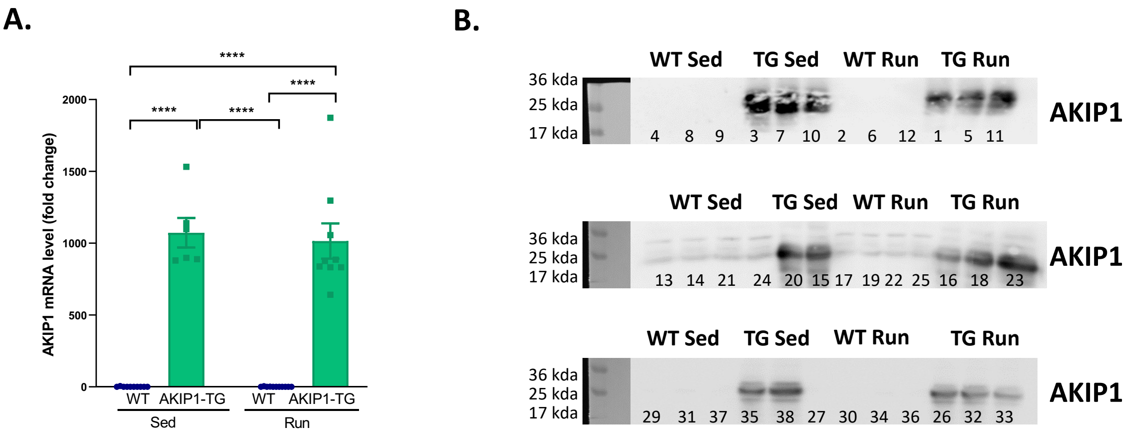

Supplementary Figure S1. Shown are *(A)* mRNA expression of AKIP1 in all four groups and *(B)* Western blot showing protein levels of AKIP1 in all four groups. Full and original Western blots are included in Supplementary Figures. Mice included were aged 8-12 weeks old. WT= wild type mice, AKIP1-TG= AKIP1 transgenic mice, Sed= Sedentary, Run= Running. Graphs represent mean ± standard error of the mean (SEM). Statistical analysis was performed with Two-way ANOVA and *post hoc* Tukey's test, <0.05 was considered statistically significant. p****<0.0001.

Supplementary Figure S2. Desmosome staining to visualize cardiomyocyte length and width.

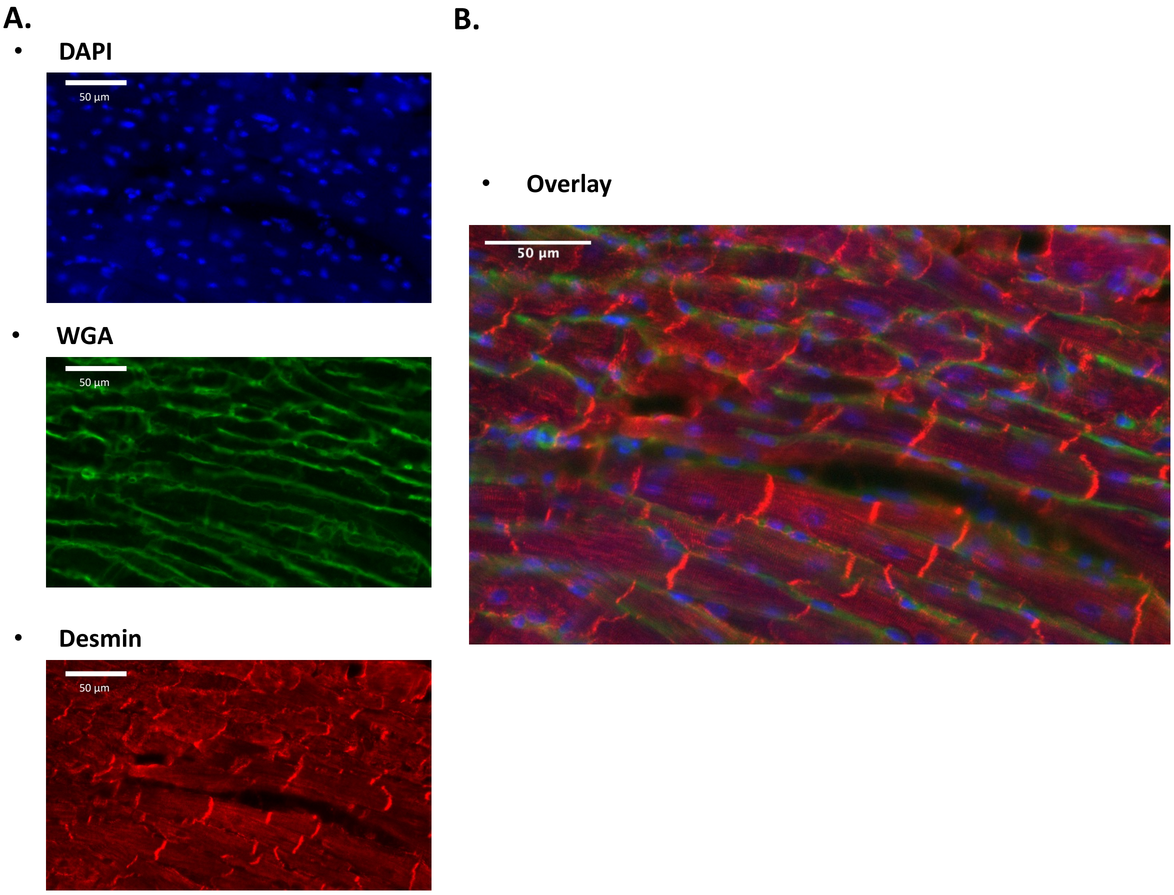

Supplementary Figure S2. 4',6-diamidino-2-phenylindole (DAPI), Wheat germ agglutinin (WGA) and Desmin staining to measure cardiomyocyte length and width. Shown are *(A)* images of individual channels, and in *(B)* an overlay of all three channels.

Supplementary Figure S3. Sedentary gene and protein expression of pathways involved in physiological cardiac hypertrophy.

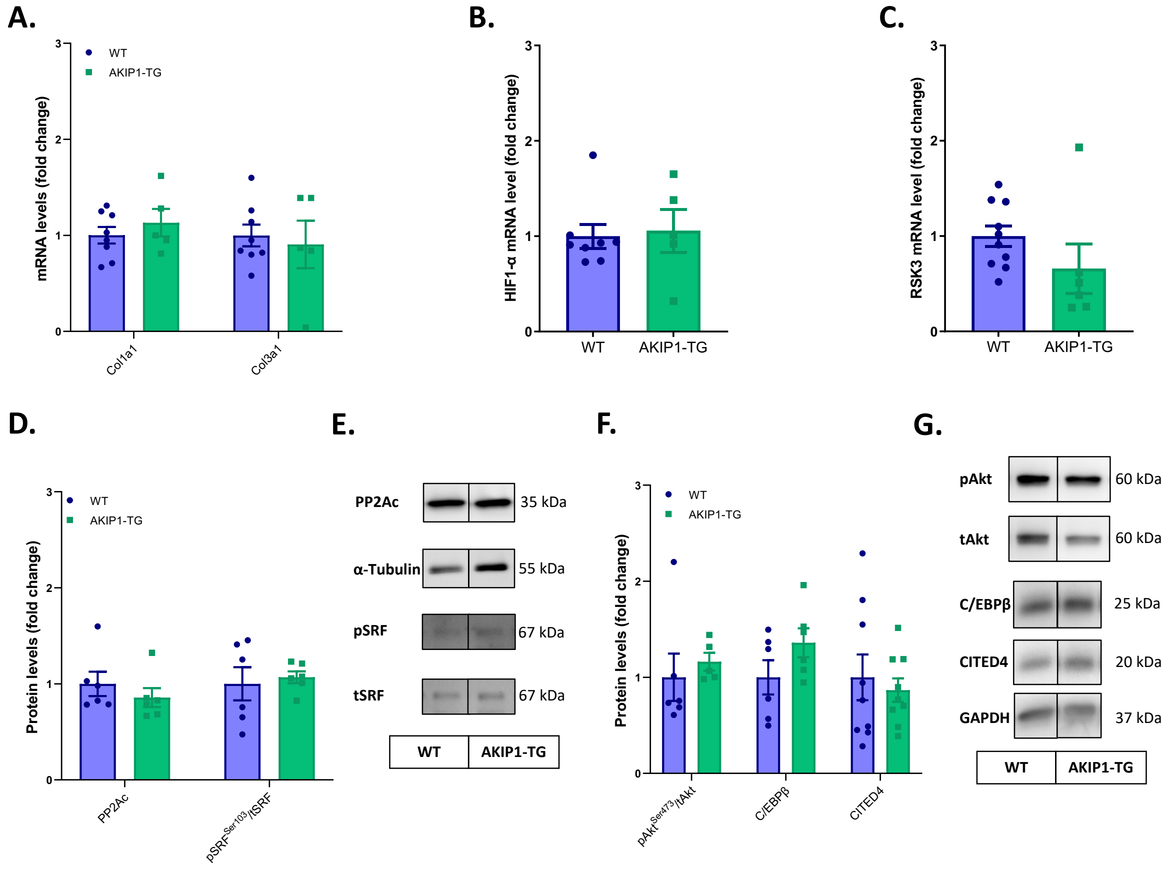

Supplementary Figure S3. Shown are *(A)* mRNA levels of collagen markers for fibrosis, N=5-8/group, *(B)* mRNA levels of hypoxia inducible factor 1 alpha (HIF1-α), a marker for angiogenesis, N=5-8/group, *(C)* mRNA level of p90 ribosomal S6 kinase 3 (RSK3), N=6-10/group, *(D)* protein levels of markers involved in the serum response factor (SRF) signalosome, N=6/group, *(E)* protein levels of the protein kinase B (Akt) pathway, N=5-9/group, *(F)* typical examples of the described Western blots. Representative Western blot images are taken from the same blot, but not always contiguous; full and original Western blots are included in Supplementary Figures. Mice included were aged 8-12 weeks old. WT= wild type mice, TG= AKIP1 transgenic mice, Col1a1= collagen type 1 alpha 1 chain, Col3a1= collagen type 3 alpha 1 chain, PP2Ac= protein phosphatase 2A catalytic subunit, α-Tubulin= alpha-tubulin, pSRF^{Ser103} = phosphorylated SRF at serine site 103, tSRF= total SRF, pAkt^{Ser473} = phosphorylated Akt at serine site 473, tAkt= total Akt, C/EBPß= CCAAT Enhancer Binding Protein Beta, CITED4= Cbp/p300 interacting transactivator with Glu/Asp rich carboxy-terminal domain 4. Graphs represent mean ± standard error of the mean (SEM). Statistical

analysis was performed with Student's *t*-test or Mann-Whitney U test, <0.05 was considered statistically significant.

Supplementary Figure S4.

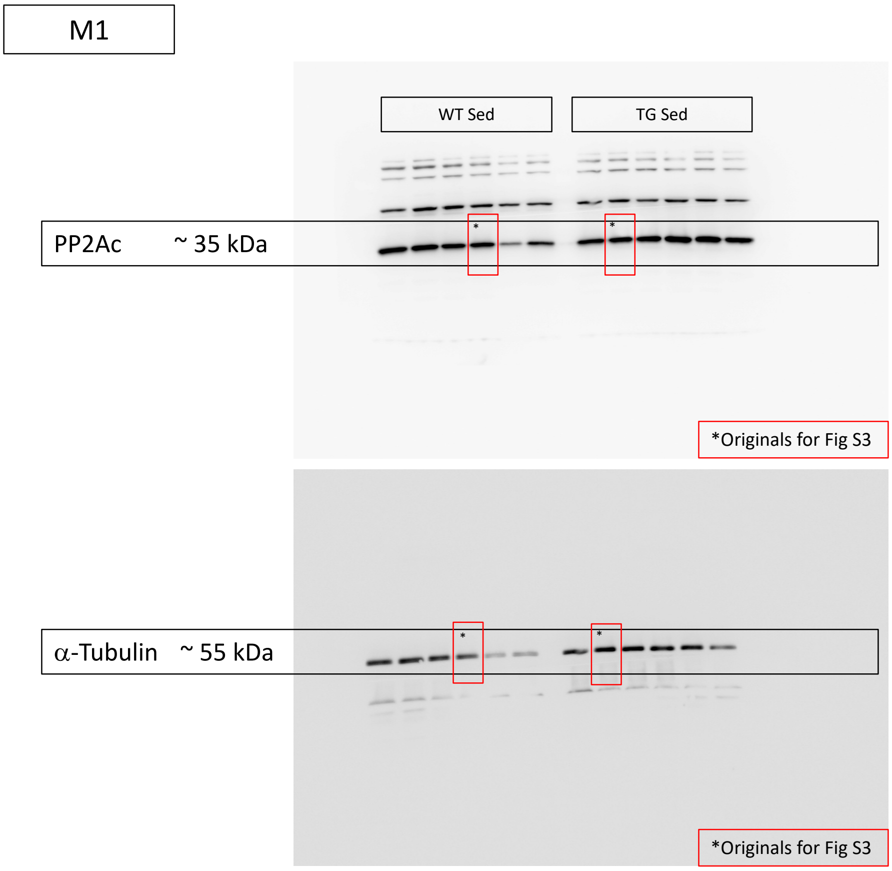

Supplementary Figure S4. Full western blot of PP2Ac and housekeeping of sedentary mice, membrane 1 (M1). AKIP1= A Kinase Interacting Protein 1, α-Tubulin= alpha tubulin, WT= wild type mice, TG= AKIP1 transgenic mice, Fig= Figure.

Supplementary Figure S5.

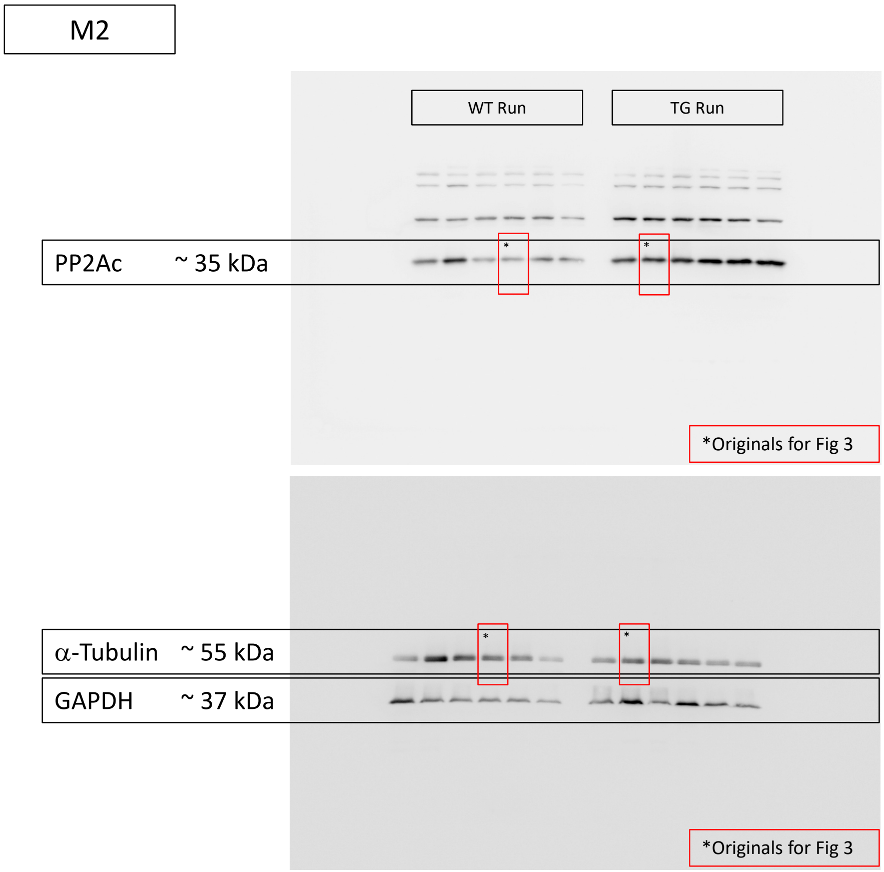

Supplementary Figure S5. Full western blot of PP2Ac and housekeeping of running mice, membrane 2 (M2). PP2Ac= protein phosphatase 2A catalytic subunit, α-Tubulin= alpha tubulin, GAPDH= glyceraldehyde 3-phosphate dehydrogenase, WT= wild type mice, TG= AKIP1 transgenic mice, Run= Running, Fig=Figure.

Supplementary Figure S6.

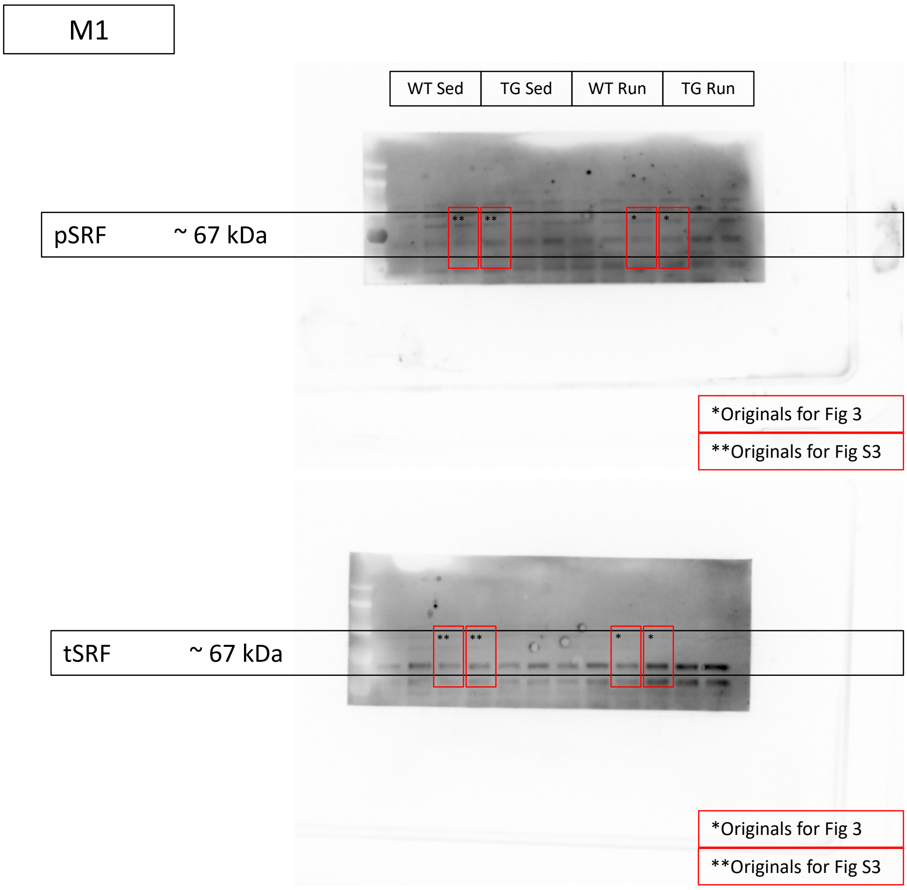

Supplementary Figure S6. Full western blot of pSRF and tSRF, membrane 1 (M1). pSRF= phosphorylated serum response factor at serine site 103, tSRF= total serum response factor, WT= wild type mice, TG= AKIP1 transgenic mice, Sed= Sedentary, Run= Running, Fig= Figure.

Supplementary Figure S7.

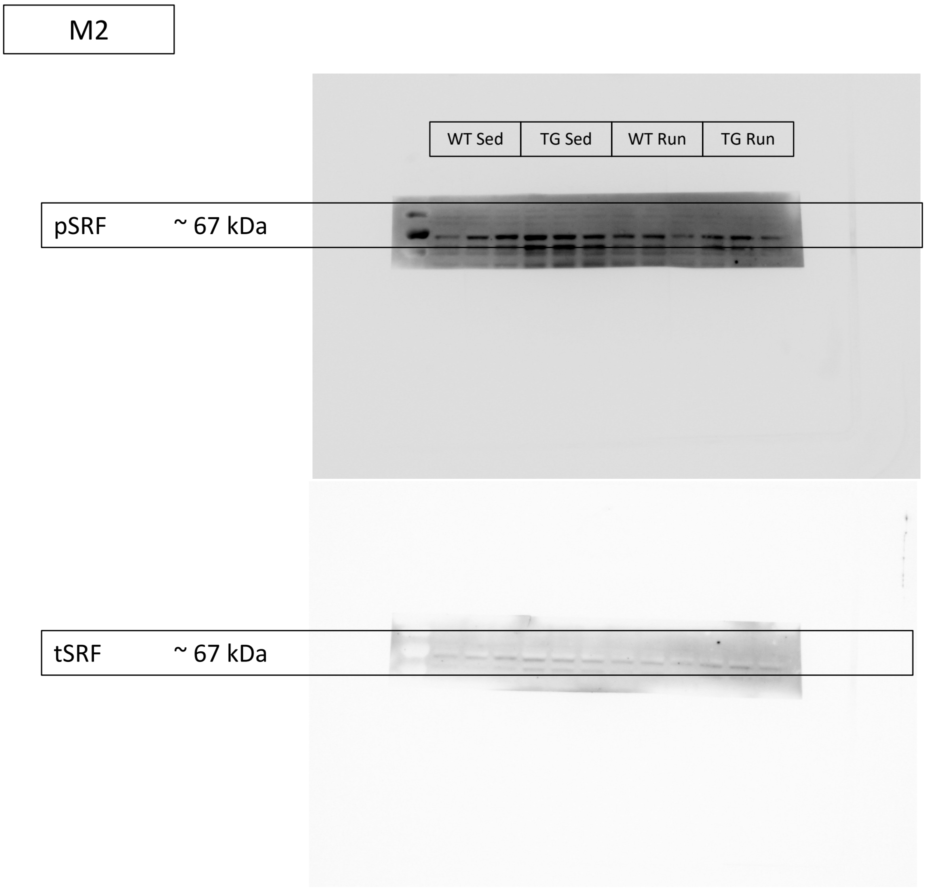

Supplementary Figure S7. Full western blot of pSRF and tSRF, membrane 2 (M2). pSRF= phosphorylated serum response factor at serine site 103, tSRF= total serum response factor, WT= wild type mice, TG= AKIP1 transgenic mice, Sed= Sedentary, Run= Running.

Supplementary Figure S8.

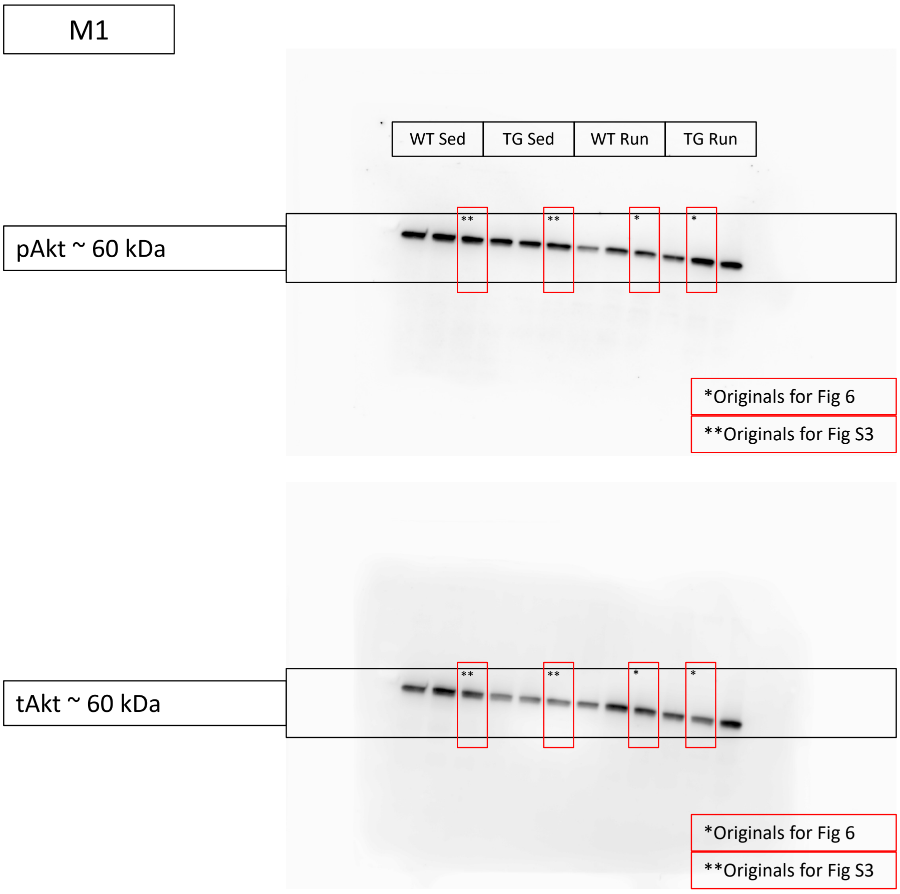

Supplementary Figure S8. Full western blot of pAkt and tAkt, membrane 1 (M1). pAkt= phosphorylated protein kinase B at serine site 473, tAkt= total protein kinase B, WT= wild type mice, TG= AKIP1 transgenic mice, Sed= Sedentary, Run= Running, Fig= Figure.

Supplementary Figure S9.

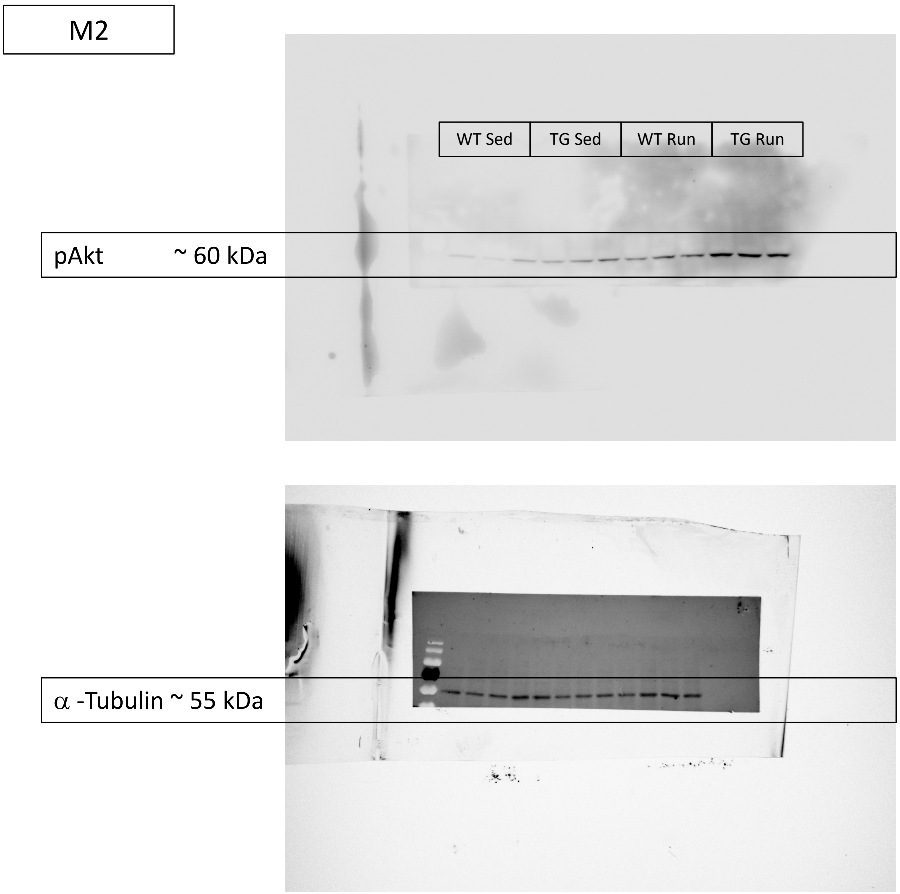

Supplementary Figure S9. Full western blot of pAkt and α-Tubulin, membrane 2 (M2). pAkt= phosphorylated protein kinase B at serine site 473, α-Tubulin= alpha tubulin, WT= wild type mice, TG= AKIP1 transgenic mice, Sed= Sedentary, Run= Running.

Supplementary Figure S10.

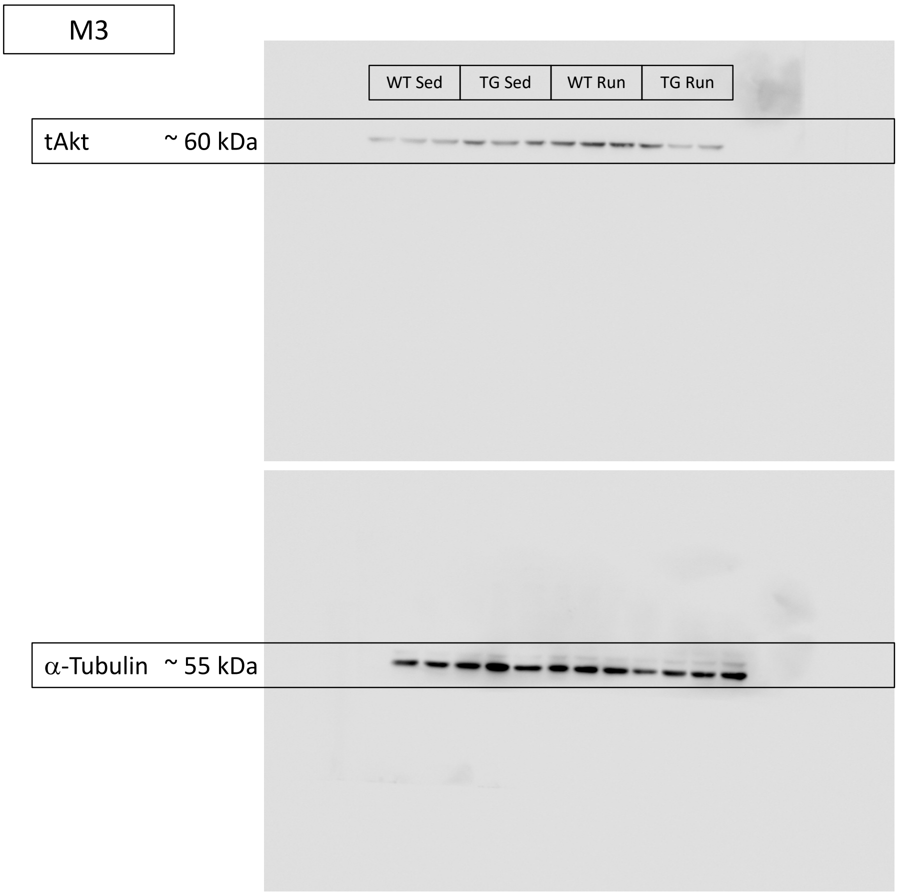

Supplementary Figure S10. Full western blot of tAkt and α-Tubulin, membrane 3 (M3). tAkt= total protein kinase B, α-Tubulin= alpha tubulin, WT= wild type mice, TG= AKIP1 transgenic mice, Sed= Sedentary, Run= Running.

Supplementary Figure S11.

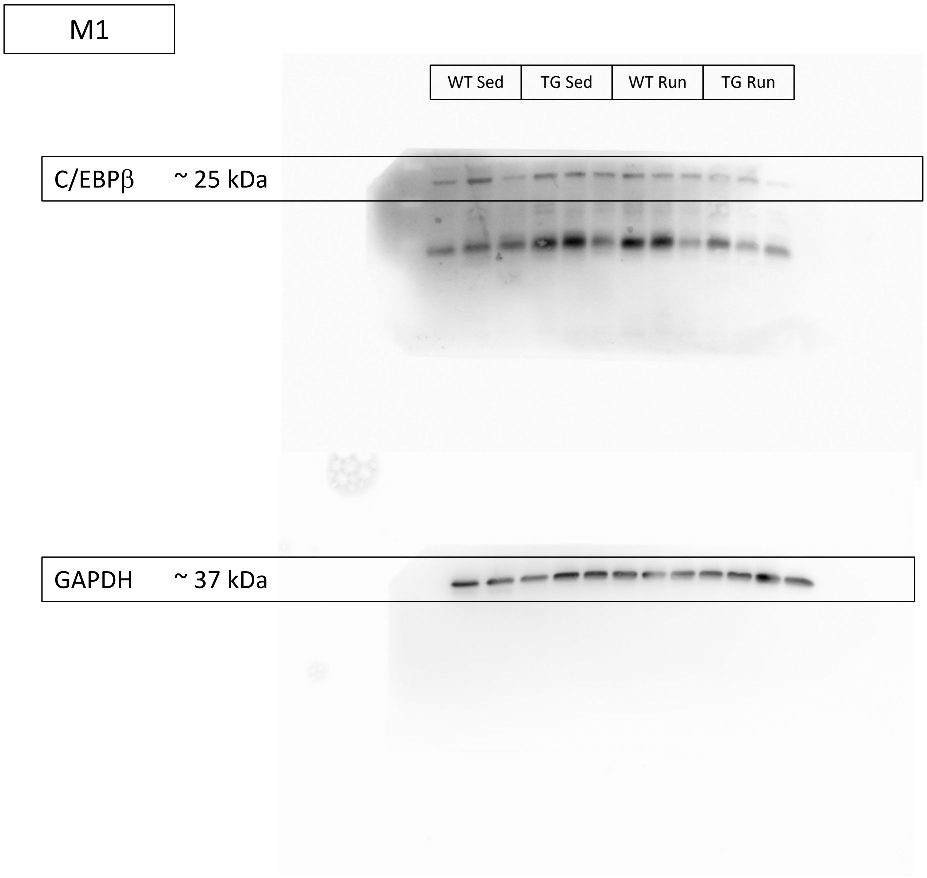

Supplementary Figure S11. Full western blot of C/EBPß and housekeeping, membrane 1 (M1), images were flipped due to backward transfer. C/EBPβ= CCAAT enhancer binding protein beta, GAPDH= glyceraldehyde 3-phosphate dehydrogenase, WT= wild type mice, TG= AKIP1 transgenic mice, Sed= Sedentary, Run= Running.

Supplementary Figure S12.

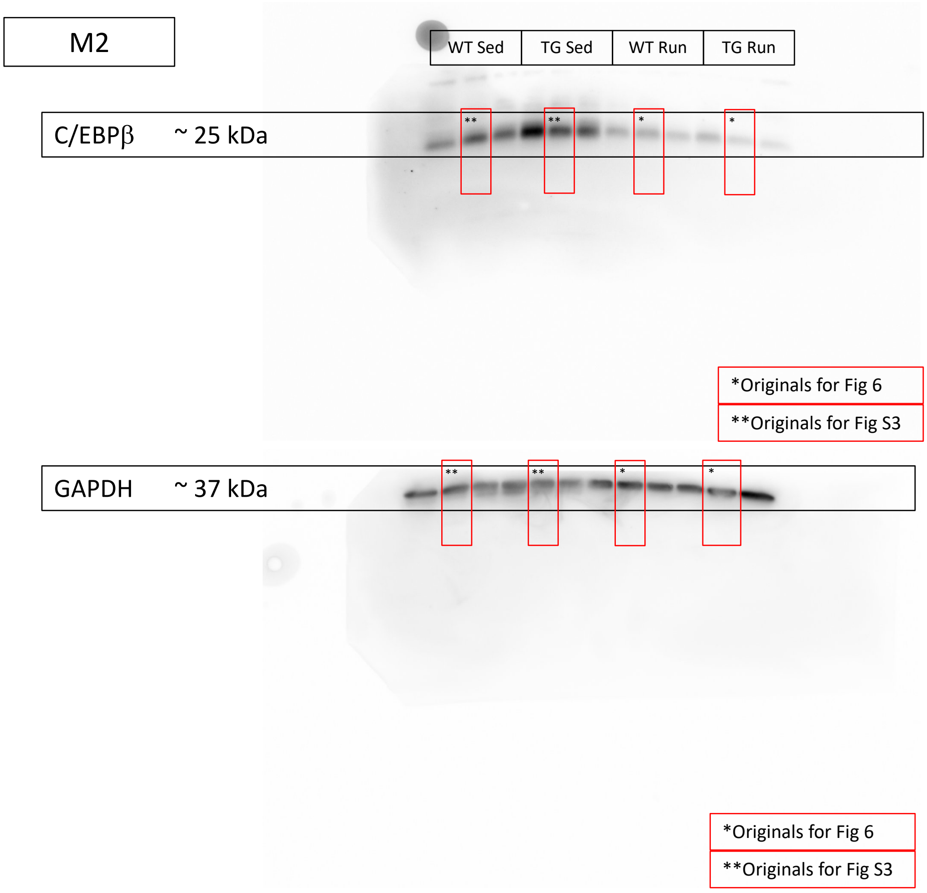

Supplementary Figure S12. Full western blot of C/EBPß and housekeeping, membrane 2 (M2), images were flipped due to backward transfer. C/EBPβ= CCAAT enhancer binding protein beta, GAPDH= glyceraldehyde 3-phosphate dehydrogenase, WT= wild type mice, TG= AKIP1 transgenic mice, Sed= Sedentary, Run= Running, Fig= Figure.

Supplementary Figure S13.

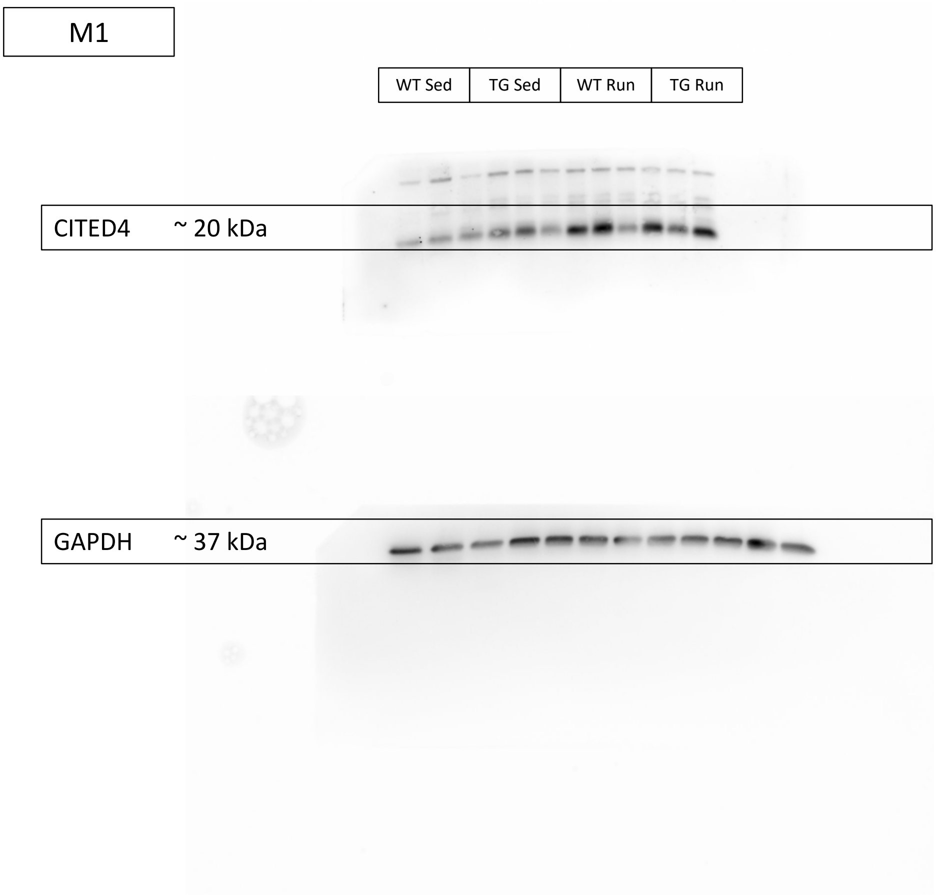

Supplementary Figure S13. Full western blot of CITED4 and housekeeping, membrane 1 (M1), images were flipped due to backward transfer. CITED4= Cbp/p300 interacting transactivator with Glu/Asp rich carboxy-terminal domain 4, GAPDH= glyceraldehyde 3-phosphate dehydrogenase, WT= wild type mice, TG= AKIP1 transgenic mice, Sed= Sedentary, Run= Running.

Supplementary Figure S14.

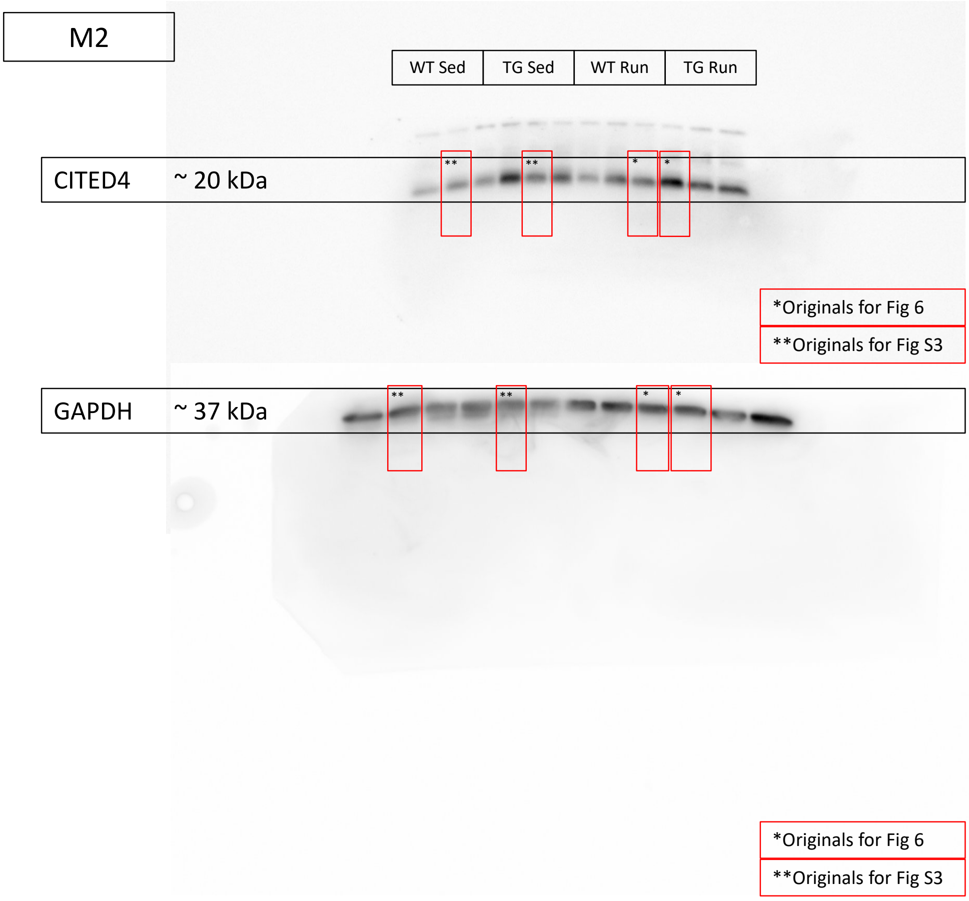

Supplementary Figure S14. Full western blot of CITED4 and housekeeping, membrane 2 (M2), images were flipped due to backward transfer. CITED4= Cbp/p300 interacting transactivator with Glu/Asp rich carboxy-terminal domain 4, GAPDH= glyceraldehyde 3-phosphate dehydrogenase, WT= wild type mice, TG= AKIP1 transgenic mice, Sed= Sedentary, Run= Running, Fig= Figure.

Supplementary Figure S15.

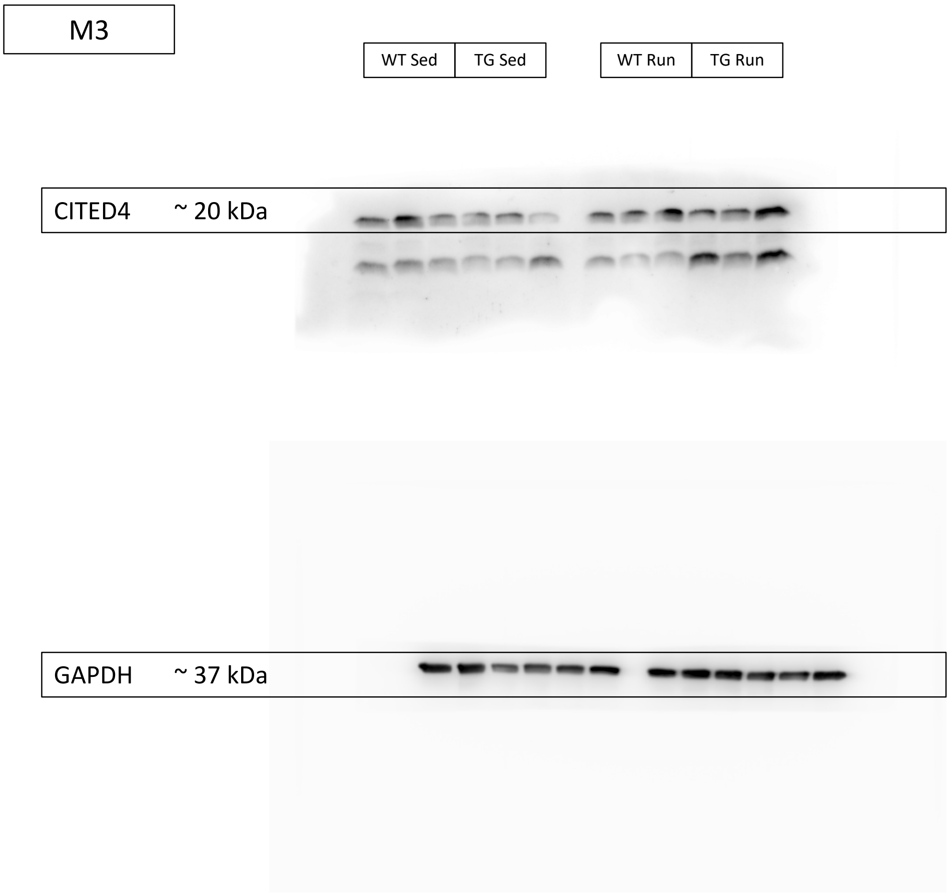

Supplementary Figure S15. Full western blot of CITED4 and housekeeping, membrane 3 (M3). CITED4= Cbp/p300 interacting transactivator with Glu/Asp rich carboxy-terminal domain 4, GAPDH= glyceraldehyde 3-phosphate dehydrogenase, WT= wild type mice, TG= AKIP1 transgenic mice, Sed= Sedentary, Run= Running.

Supplementary Figure S16.

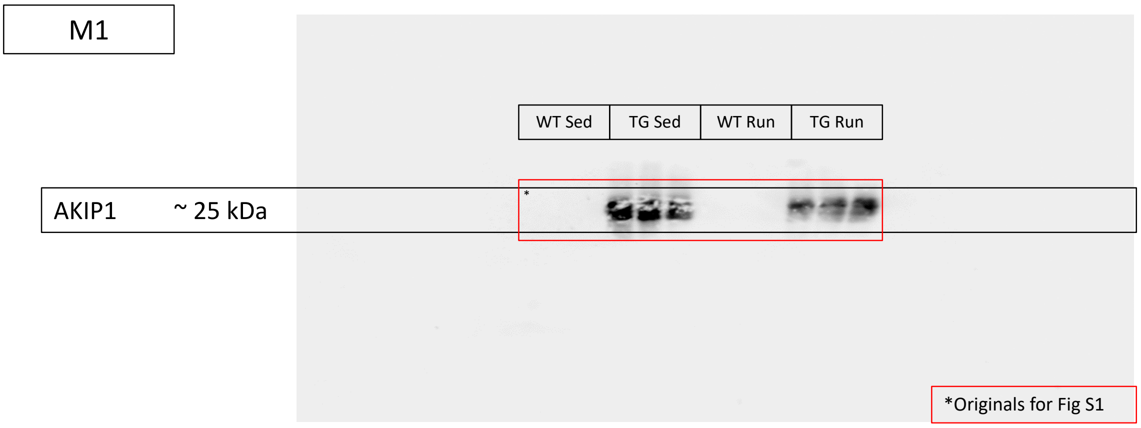

Supplementary Figure S16. Full western blot of AKIP1 and housekeeping, membrane 1 (M1). AKIP1= A Kinase Interacting Protein 1. WT= wild type mice, TG= AKIP1 transgenic mice, Sed= Sedentary, Run= Running, Fig= Figure.

Supplementary Figure S17.

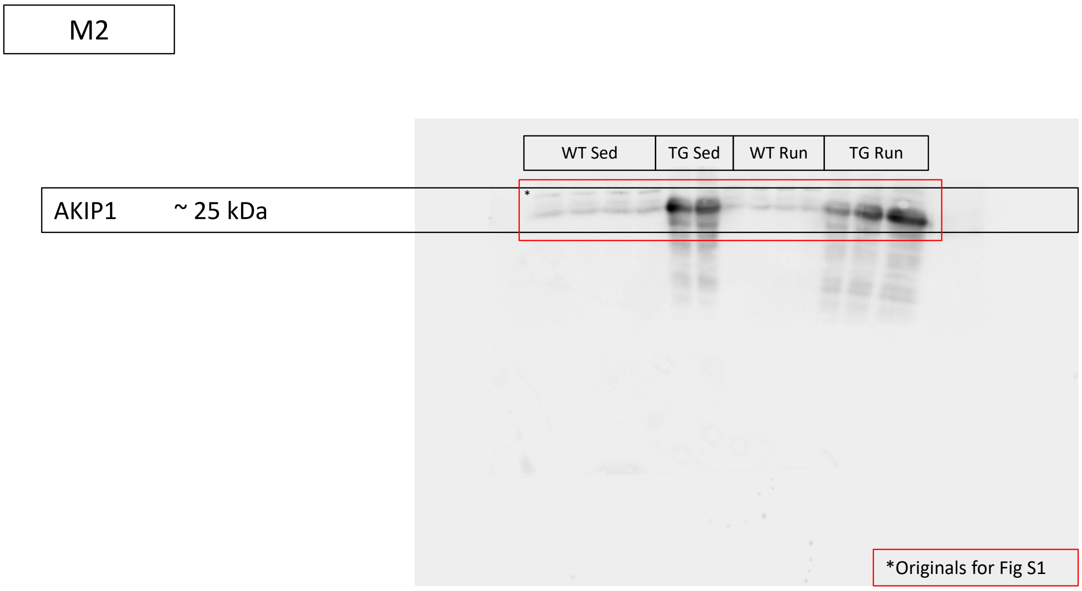

Supplementary Figure S17. Full western blot of AKIP1 and housekeeping, membrane 2 (M2). AKIP1= A Kinase Interacting Protein 1. WT= wild type mice, TG= AKIP1 transgenic mice, Sed= Sedentary, Run= Running, Fig= Figure.

Supplementary Figure S18.

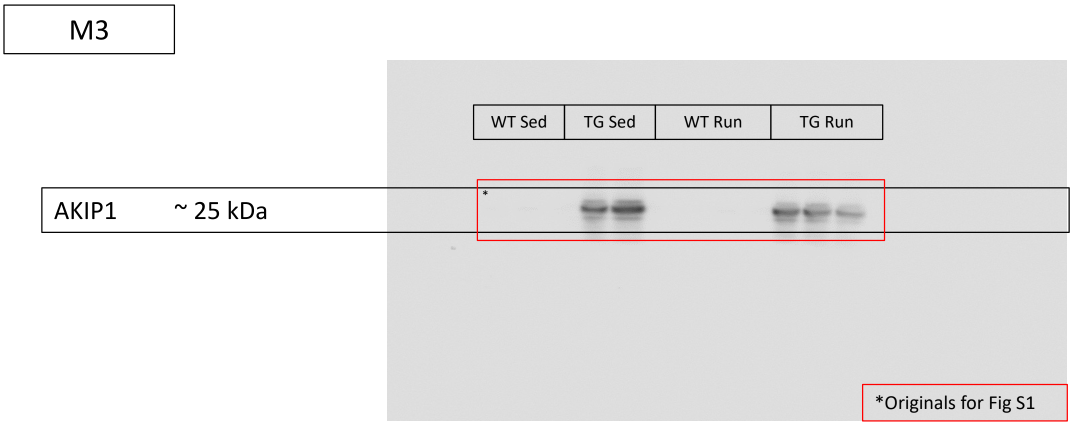

Supplementary Figure S18. Full western blot of AKIP1 and housekeeping, membrane 3 (M3). AKIP1= A Kinase Interacting Protein 1. WT= wild type mice, TG= AKIP1 transgenic mice, Sed= Sedentary, Run= Running, Fig= Figure.

References

1. Yu, H. *et al.* AKIP1, a Cardiac Hypertrophy Induced Protein that Stimulates Cardiomyocyte Growth via the Akt Pathway. *Int J Mol Sci* **14**, 21378–21393 (2013).
